# ARID1A Governs Genomic Stability and Proliferation in SCLC via c-MYC/PARP1 Suppression Driving Vulnerability to BET Inhibitors

**DOI:** 10.34133/research.0908

**Published:** 2025-10-02

**Authors:** Guozhen Cao, Liying Ma, Xueqin Dai, Peng Hou, Xinhuang Yao, Gongfeng Li, Jiahui Zhang, Ceshi Chen, Wenchu Lin

**Affiliations:** ^1^ The Second Affiliated Hospital, School of Medicine, The Chinese University of Hong Kong, Shenzhen & Longgang District People’s Hospital of Shenzhen, Shenzhen 518172, P. R. China.; ^2^ University of Science and Technology of China, Hefei 230036, Anhui, P. R. China.; ^3^ High Magnetic Field Laboratory, Chinese Academy of Sciences, Hefei 230031, Anhui, P. R. China.; ^4^ Yunnan Key Laboratory of Breast Cancer Precision Medicine, Academy of Biomedical Engineering, Kunming Medical University, Kunming 650500, Yunnan, P. R. China.; ^5^ Yunnan Key Laboratory of Breast Cancer Precision Medicine, Yunnan Cancer Hospital, The Third Affiliated Hospital of Kunming Medical University, Yunnan Hospital of Peking University Cancer Hospital, Kunming 650118, Yunnan, P. R. China.

## Abstract

Small cell lung cancer (SCLC) is a highly aggressive neuroendocrine tumor among the most lethal cancers. ARID1A has a dual role in oncogenic and tumor-suppressive functions, depending on the type of cancer. However, its role in SCLC remains unclear. Herein, we showed that ARID1A was highly expressed and correlated with prognosis in SCLC. In vitro and in vivo investigations manifested that *ARID1A* inhibited cell survival, proliferation, and tumor growth, functioning as a gatekeeper for cell proliferation and a caretaker of genome stability in SCLC cells. Mechanistically, ARID1A transcriptionally represses *c-MYC* and *PARP1* expression. ARID1A depletion triggered replication stress response (RSR), DNA double-strand breaks (DSBs), and PI3K/AKT pathway activation, which could be counteracted by *c-MYC* or *PARP1* silencing. These findings establish ARID1A as a critical antagonist of c-MYC and PARP1 signaling, coordinating proliferation control and genomic integrity maintenance. Furthermore, we revealed that *ARID1A* loss confers therapeutic vulnerability to the BET inhibitor (JQ1). The ARID1A-targeting compound BRD-K98645985 exhibited potent single-agent antitumor activity and synergized with JQ1 to suppress SCLC progression, highlighting a novel combinatorial therapeutic strategy. Collectively, our findings elucidate ARID1A as a critical regulator of SCLC pathogenesis through its dual control of proliferation and genomic stability while revealing novel therapeutic vulnerabilities that can be exploited through ARID1A-targeting strategies and BET inhibitor combinations.

## Introduction

Small cell lung cancer (SCLC) is one of the most aggressive malignancies, representing approximately 10% to 15% of all new lung cancer cases worldwide [1,2]. SCLC is characterized by neuroendocrine features, high metastatic potential, and rapid development of chemoresistance [3]. Approximately two-thirds of SCLC patients are diagnosed at an extensive stage with limited treatment options, and the prognosis is very poor. Identifying the potential therapeutic targets and developing promising novel treatment strategies have been severely impeded due to the lack of knowledge about the biology and pathogenesis of SCLC.

AT-rich interaction domain-containing protein 1A (ARID1A) encodes a signature subunit of the adenosine triphosphate (ATP)-dependent chromatin remodeling complex BAF (Brahma associated factor). *ARID1A* is recurrently mutated in a broad spectrum of human malignancies, with the highest mutation rate in the clear cell type of ovarian cancer. Additionally, *ARID1A* is frequently monoallelically mutated [4–6]. Interestingly, several recent studies showed that low *ARID1A* expression is associated with a poor prognosis in gastric [7–9], breast [10,11], and urothelial bladder cancers [12]. Therefore, *ARID1A* alterations caused by mutation and low expression might be essential in tumor progression [4]. The experimental investigations of ARID1A inactivation in cellular and mouse models have revealed that ARID1A acts as a tumor suppressor during the initiation and progression stages of carcinogenesis in gynecological [13], liver [14], and colorectal cancer models [15]. However, ARID1A also possesses an oncogenic function in an Apc-deficient setting and Ras-induced pancreatic tumors [16]. The roles of ARID1A might be context dependent [14].

Molecular mechanisms related to the tumor suppressor role of ARID1A seem to be distinct among different cancerous tumors. Inactivation of *ARID1A* might perturb enhancer-controlled gene expression in specific tissue settings, promoting cell proliferation [17]. For example, ARID1A directly binds to the *TERT* promoter region and suppresses *TERT* expression by promoting chromatin condensation in ovarian clear cell carcinoma [18,19]. DNA damage response (DDR) occurs in the chromatin context. ARID1A-mediated chromatin remodeling involves repairing DNA damage, including mismatch and DNA double-strand break (DSB) repair [20]. An essential DNA damage sensor, ATR recruits ARID1A to DSB sites, facilitating the DNA damage repair process [11,21]. Another study indicates that ARID1A associates with p53 and co-regulates p53 targets in uterine endometrioid and ovarian clear cell carcinoma [21,22]. Therefore, the mechanisms by which ARID1A exerts its functions might depend on tissue context. Understanding the molecular mechanisms underlying tumor suppression or oncogenesis by ARID1A could facilitate the discovery of vulnerabilities to be harnessed therapeutically. Several studies have demonstrated that small inhibitors targeting PARP [11], HSP90, ATR [23,24], EZH2 [25], and HDAC6 [26] could lead to synthetic lethality in the *ARID1A*-deficient tumors. However, the roles of ARID1A are poorly defined in SCLC.

In the present study, we first performed an integrated analysis of *ARID1A* mutation, expression, and correlation with clinical data. Then, in vitro and in vivo investigations using knockdown (KD) and overexpression approaches highlighted ARID1A’s function as a tumor suppressor in SCLC. Furthermore, we utilized SCLC cell line and mouse models to elucidate the consequences of *ARID1A* loss or gain, and discovered a mechanism by which ARID1A suppresses cell proliferation by modulating c-MYC expression and safeguards genome integrity by suppressing PARP1. Finally, we unveiled that *ARID1A* loss constituted vulnerabilities to be harnessed therapeutically by BET inhibitors, while the ARID1A-targeting compound BRD-K98645985 showed potent single-agent activity and synergistic efficacy with JQ1, revealing a novel combinatorial strategy against SCLC. Thus, our results provide biological insights into the function of ARID1A as a tumor suppressor in SCLC and present a therapeutic strategy to target SCLC with high ARID1A expression.

## Results

### ARID1A expression is elevated in SCLC and positively associated with SCLC patient survival

**Fig. 1.**
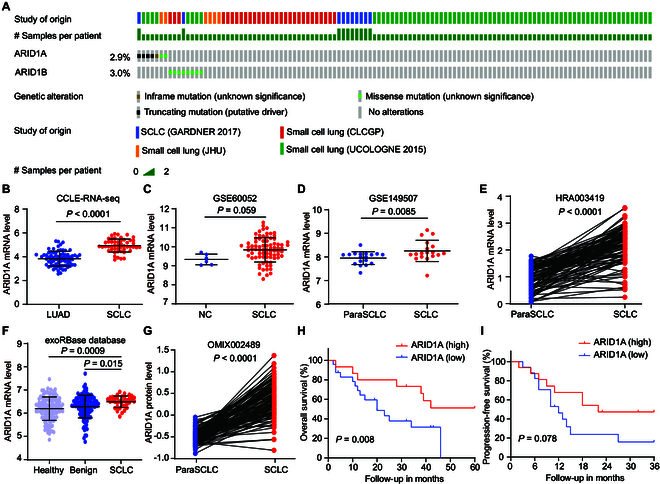
Characterization of mutation rate and expression of *ARID1A* in SCLC. (A) Oncoprint of *ARID1A* and *ARID1B* mutations in human primary SCLC. (B to F) Scatterplots of *ARID1A* expression in SCLC cell lines relative to LUAD cell lines (B; CCLE dataset), in primary SCLC relative to normal lung tissues (C; GEO60052 dataset), in SCLC tissues relative to adjacent normal tissues (paraSCLC) (D; GEO149507 dataset), in 107 paired SCLC and paraSCLC retrieved from the GSA database (E; HRA003419 dataset), and in SCLC blood relative to healthy and benign blood from exoRBase database 2.0 (F). (G) Protein expression of ARID1A in 112 paired SCLC and paraSCLC, retrieved from the OMIX database (OMIX002489). (H and I) Kaplan–Meier survival analysis of the correlations between ARID1A expression and OS (H) and PFS (I) in 41 (H) or 33 (I) patients with SCLC by log-rank tests, respectively. Statistical analysis was performed using 2-tailed unpaired Student’s *t* tests for (B) to (E), and 2-tailed paired Student’s *t* tests for (F) and (G).

To gain insight into the role of ARID1A in SCLC, we first investigated the somatic mutation profiles of *ARID1A* and *ARID1B* in 249 SCLC clinical specimens obtained from the cBioPortal database (https://www.cbioportal.org/). Unlike ovarian clear cell carcinoma, cervical adenocarcinomas, and gastric carcinoma, SCLC harbored a relatively low frequency of *ARID1A* and *ARID1B* mutations (Fig. 1A). Characterization of the mutation spectrum of *ARID1A* and *ARID1B* in 52 SCLC cell lines also indicated relatively low mutation rates for both ARID1s (Fig. S1A). We then performed a comparative analysis of *ARID1A* expression in multiple RNA-sequencing (RNA-seq) data and microarray datasets. Compared to lung adenocarcinoma (LUAD) cell lines, SCLC cell lines display markedly higher *ARID1A* expression (Fig. 1B and Fig. S1B). In addition, we found that *ARID1A* expression was relatively higher in primary SCLC samples (66 SCLC samples) than normal lung tissues (Fig. 1C). Similarly, *ARID1A* mRNA expression was significantly elevated in SCLC tissues compared to both adjacent noncancerous tissues (Fig. 1D and E) and blood samples from healthy individuals or patients with benign conditions (Fig. 1F). Consistently, the protein levels of ARID1A in SCLC tissues were found to be higher than in adjacent noncancerous tissues (Fig. 1G). Furthermore, a clinical correlation analysis was performed to address the prognostic significance of *ARID1A* expression. Patients with high *ARID1A* expression had a better overall survival (OS) (median survival of 48 months of the high *ARID1A* group versus 20 months of the low *ARID1A* group, *P* = 0.008, *n* = 41, log-rank test) (Fig. 1H) and progression-free survival (PFS) (median survival of 22 months of the high *ARID1A* group versus 12 months of the low *ARID1A* group, *P* = 0.078, *n* = 33, log-rank test) (Fig. 1I). These data support that *ARID1A* expression could serve as a novel prognostic marker for SCLC. Together, these data indicate that *ARID1A* exhibits a low somatic mutation rate and high *ARID1A* expression, offering a survival advantage for SCLC patients.

### High ARID1A expression suppresses cell proliferation in SCLC cells

**Fig. 2.**
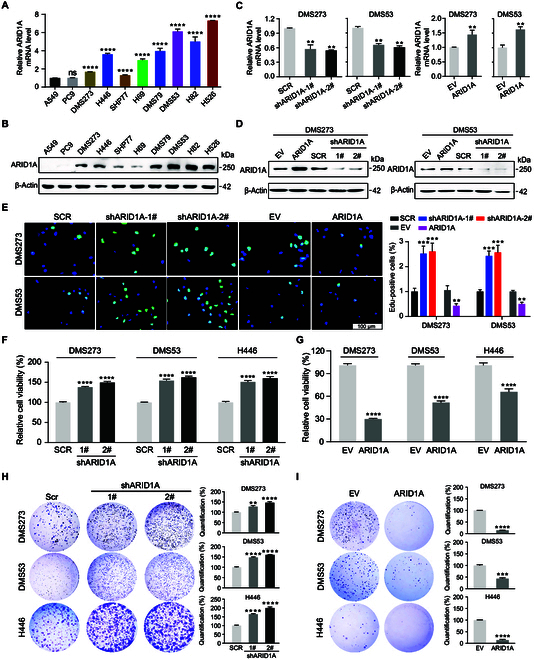
ARID1A suppresses cell viability and clonogenicity in SCLC. (A) RT-qPCR analysis of *ARID1A* expression in 8 SCLC cell lines and 2 LUAD cell lines. A549 was used as the control for standardization. (B) Detection of ARID1A expression by Western blot across a panel of lung cancer cell lines. β-Actin was used as a loading control. (C) RT-qPCR analysis of *ARID1A* in DMS273 and DMS53 cells with *ARID1A* KD and overexpression (OE). (D) Western blot analysis of ARID1A expression following *ARID1A* KD and OE in DMS273 and DMS53 cells. (E) Representative images of EdU staining in SCLC cells following *ARID1A* KD and OE (left). Quantitative analysis of the results is shown on the right. (F to I) Cell viability (F and G) and colony formation (H and I) assay after *ARID1A* KD (F and H)/OE (G and I). The quantitative analysis of the colonies was performed using ImageJ software. Data are shown as the mean ± SEM, *n* ≥ 3 independent experiments. Statistical analysis was performed using 2-tailed unpaired Student’s *t* tests. ns, no significance; **P < 0.01, ***P < 0.001, ****P < 0.0001.

A series of biochemical and molecular experiments was carried out to determine the biological functions of ARID1A in SCLC. We first analyzed the expression of *ARID1A* in a panel of SCLC cell lines at the mRNA and protein levels by reverse transcription quantitative polymerase chain reaction (RT-qPCR) and Western blot. RT-qPCR analysis validated a higher *ARID1A* expression in SCLC cells than in LUAD cells (Fig. 2A). Western blot assays also confirmed that SCLC cells expressed much higher ARID1A than did LUAD cells (Fig. 2B). We then generated stable cell lines with *ARID1A* KD and overexpression (Fig. 2C and D and Fig. S2A). EdU staining assay showed that *ARID1A* depletion promoted cell proliferation in DMS273 and DMS53 cells. Complementarily, cell proliferation was significantly decreased compared with control cells once ectopic *ARID1A* was overexpressed (Fig. 2E). Next, cell viability test using the CellTiter-Glo assay demonstrated that *ARID1A* enhanced cell survival in SCLC cells examined (Fig. 2F and G). Furthermore, long-term colony formation assays demonstrated that the colony formation rate was significantly increased upon *ARID1A* depletion and decreased once *ARID1A* was overexpressed (Fig. 2H and I). Apoptosis measurement by flow cytometry using annexin V staining demonstrated no statistical difference in the induction of apoptosis between control cells and *ARID1A*-defective cells (Fig. S2B and C). Consistently, *ARID1A* overexpression in SCLC cells had minimal effect on apoptosis (Fig. S2D and E). Together, these data indicate that ARID1A is indispensable for the cell survival, proliferation, and clonogenicity of SCLC cells.

### ARID1A inhibits SCLC tumor growth in vivo

**Fig. 3.**
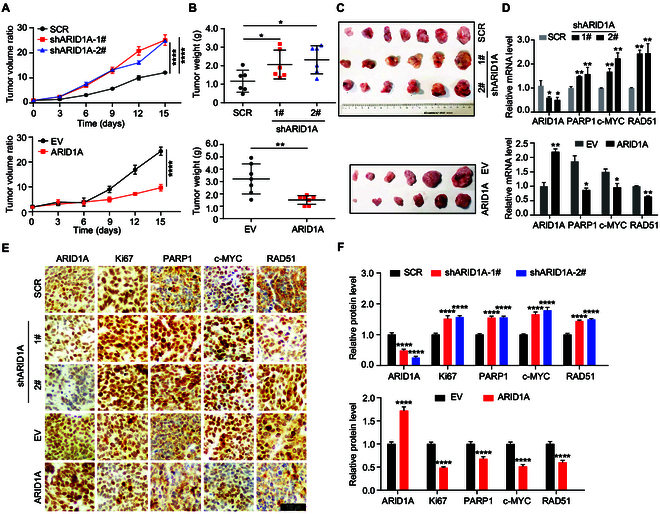
ARID1A restrains tumor growth in vivo*.* (A) Tumor volume curves of DMS273 control cells and *ARID1A* depletion cells (top) or overexpression (bottom). (B) Scatterplot representing tumor weights of DMS273 control cells and *ARID1A* depletion cells (top) or overexpression (bottom) at the endpoint of experiments. (C) Imaging of representative tumors from each group. (D) RT-qPCR analysis of mRNA expression of *ARID1A*, *c-MYC*, *PARP1*, and *RAD51* after KD (top)/OE (bottom) of *ARID1A* in vivo. (E) IHC analysis of the expression of ARID1A, c-MYC, PARP1, RAD51, and γH2AX in subcutaneous tumor tissues of mice. Scale bars, 100 μm. (F) Quantification of the IHC. The signals were quantified by IOD, equal to optical density by area, by using ImageJ software and indicated as mean ± SEM. Statistical analysis was performed using 2-tailed unpaired Student’s *t* tests. *P < 0.05, **P < 0.01, ****P < 0.0001.

To further elucidate the tumor-suppressing effect of ARID1A in SCLC in vivo, we assessed the effects of ARID1A on tumor progression in SCLC xenograft models. DMS273 cells carrying nontargeting (SCR) or sh*ARID1A* were injected subcutaneously into nude mice to elicit the formation of solid tumors. *ARID1A* silencing significantly promoted tumor growth, as evidenced by a significant increase in the tumor volume and weight (top panels, Fig. 3A to C). By RT-qPCR analysis, xenograft tumors showed consistent expression patterns of *PARP1*, *c-MYC*, and *RAD51* (top panel, Fig. 3D). Immunohistochemistry (IHC) staining further confirmed down-regulated ARID1A expression and up-regulation of PARP1 and c-MYC in the *ARID1A* KD group (Fig. 3E and F). As expected, increased cell proliferation was determined by IHC staining of Ki67 upon *ARID1A* depletion (Fig. 3E and F). Conversely, when mice were fed with doxycycline (Dox) to induce *ARID1A* expression, the growth of *ARID1A*-overexpressed DMS273 xenografts was significantly suppressed, resulting in reduced tumor volume, weight, and size (bottom panels, Fig. 3A to C). RT-qPCR and IHC analysis demonstrated that *ARID1A* overexpression in vivo led to the opposite of *ARID1A* depletion on cell proliferation marker, the expression of PARP1, c-MYC, and RAD51 (Fig. 3D to F). Importantly, we obtained consistent results in the H446 cell line, where no significant difference in weight loss or animal mortality was observed (Fig. S3A to F). Collectively, these comprehensive data provide robust confirmation that ARID1A serves as a potent suppressor of SCLC tumor growth in vivo.

### ARID1A suppresses cell proliferation and promotes genome stability through transcriptional regulation of *c-MYC*/*PARP1*

**Fig. 4.**
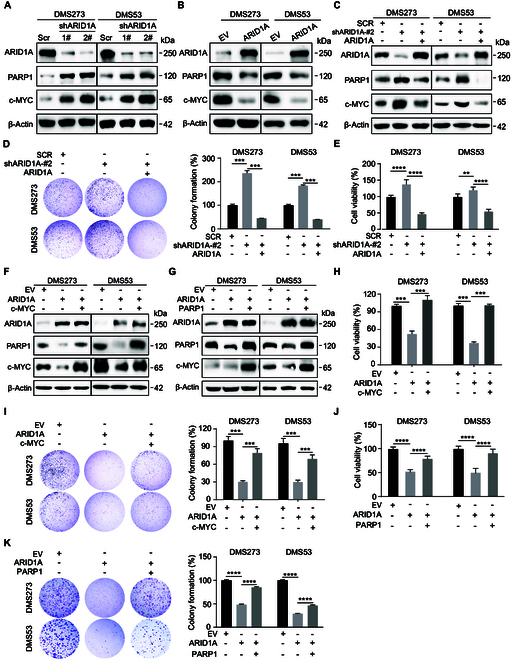
ARID1A controls SCLC cell survival by inhibiting *c-MYC*/*PARP1* expression. (A and B) Western blot analysis of the indicated proteins in DMS273 and DMS53 cells following *ARID1A* KD (A) or OE (B). (C) Western blot analysis of the indicated proteins following *ARID1A* overexpression in *ARID1A* KD DMS273 and DMS53 cells. (D and E) Clonal formation (D) and cell viability (E) assays following *ARID1A* overexpression in *ARID1A* KD DMS273 and DMS53 cells. (F and G) Western blot analysis of the indicated proteins following *c-MYC* (F) or *PARP1* (G) overexpression in *ARID1A*-overexpressing DMS273 and DMS53 cells. (H to K) Cell viability (H and J) and clonal formation (I and K) assays following *c-MYC* (H and I) or *PARP1* (J and K) overexpression in *ARID1A*-overexpressing DMS273 and DMS53 cells. Data are shown as the mean ± SEM; *n* ≥ 3 independent experiments. Statistical analysis was performed using a one-way ANOVA. **P < 0.01, ***P < 0.001, ****P < 0.0001.

It has been reported that ARID1A is a caretaker protein required to maintain genome integrity [27]. However, how ARID1A is involved in preserving genomic integrity still needs to be determined. Therefore, we sought to determine whether ARID1A influences the expression of PARP1 and c-MYC, 2 well-characterized proteins involved in the modulation of cell proliferation and genome stability. Targeting *ARID1A* with small interfering RNAs (siRNAs) led to a dramatic increase in PARP1 and c-MYC expression (Fig. S4A). The same results as siRNA-mediated *ARID1A* KD were observed in SCLC cell lines with stable *ARID1A* KD (Fig. 4A and Fig. S4B). In contrast, ectopic *ARID1A* expression suppressed c-MYC and PARP1 expressions (Fig. 4B and Fig. S4B). Importantly, ectopic expression of *ARID1A* effectively suppressed the induction of PARP1 and c-MYC, as well as inhibited cell viability and clonogenic capacity in *ARID1A* KD cells. (Fig. 4C to E). These data indicate that cell phenotypes and the regulation of PARP1 and c-MYC are *ARID1A*-dependent. To test the hypothesis that ARID1A regulates SCLC proliferation and genome stability through c-MYC and PARP1 modulation, we performed cell viability and clonogenic assays in *ARID1A*-overexpressing SCLC cells with ectopic *c-MYC* or *PARP1* expression. Our results demonstrated that *c-MYC* overexpression partially restored the proliferation and colony formation capabilities impaired by ARID1A in both DMS273 and DMS53 cell lines (Fig. 4F, H, and I). Similarly, *PARP1* overexpression also showed partial rescue effects on *ARID1A*-mediated phenotypic changes (Fig. 4G, J, and K). These findings collectively indicate that ARID1A indeed regulates SCLC cell survival and clonogenic potential through its effects on c-MYC and PARP1 expression.

**Fig. 5.**
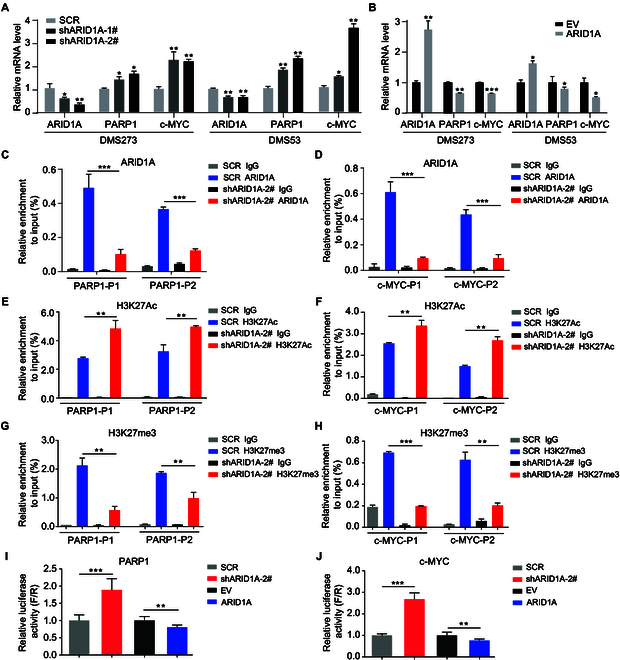
ARID1A transcriptionally suppresses the expression of *c-MYC* and *PARP1*. (A and B) RT-qPCR analysis of *c-MYC* and *PARP1* mRNA expression in DMS273 and DMS53 cells following *ARID1A* KD (A) or OE (B). (C and D) ChIP-PCR analysis of DMS273 cells expressing control or sh*ARID1A* using antibodies against ARID1A for the promoter regions of *PARP1* (C) and *c-MYC* (D). (E to H) ChIP-PCR analysis of DMS273 cells expressing control or sh*ARID1A* using antibodies against H3K27Ac (E and F) and H3K27me3 (G and H) for the promoter regions of *PARP1* (E and G) and *c-MYC* (F and H). (I and J) The relative luciferase activities were detected in DMS273 cells with *ARID1A* KD or OE after transfection with the PARP1 promoter-driven luciferase (I) or the *c-MYC* promoter-driven luciferase (J) constructs. The firefly luciferase activities were measured and normalized to Renilla luciferase activities (F/R). Data are shown as the mean ± SEM; *n* ≥ 3 independent experiments. Statistical analysis was performed using 2-tailed unpaired Student’s *t* tests. *P < 0.05, **P < 0.01, ***P < 0.001.

Mechanistically, RT-qPCR analysis revealed that KD and overexpression of *ARID1A* modulated the expression of *PARP1* and *c-MYC* at the mRNA level (Fig. 5A and B). Analysis of data from the Cancer Cell Line Encyclopedia (CCLE) demonstrated a statistically significant inverse correlation between *ARID1A* and *c-MYC* expression levels at the transcriptional level (Fig. S4C). The expression pattern of *PARP1* and *c-MYC* is consistent with the observation in Western blot analysis (Fig. 4A and B). To provide mechanistic evidence of the potential ARID1A transcriptional regulation of *c-MYC* and *PARP1*, the chromatin immunoprecipitation (ChIP) assay was used to evaluate the association of ARID1A with the *c-MYC* and *PARP1* promoter regions. qPCR analysis after ChIP indicated that ARID1A could bind to the promoters of *PARP1* and *c-MYC* (Fig. 5C and D). After being treated with *ARID1A* shRNA, ARID1A occupancy on the promoters of *PARP1* and *c-MYC* was significantly diminished (Fig. 5C and D). Concomitantly, the elevated H3K27Ac (Fig. 5E and F) and reduced H3K27me3 (Fig. 5G and H) at the promoters of *c-MYC* and *PARP1* were observed. These data confirmed that ARID1A inhibits gene expression by binding to the promoters of *c-MYC* and *PARP1* and impacting chromatin structure. To further verify that *c-MYC* and *PARP1* are the direct targets of ARID1A, we constructed luciferase reporter gene plasmids where the linear sequence in the putative binding sites for ARID1A of *c-MYC* and *PARP1* was fused to the pGL4 plasmid of luciferase. The dual-luciferase reporter assays found that *ARID1A* KD significantly up-regulated the activity of luciferase reporter genes placed under the control of the promoter of *c-MYC* or *PARP1* (Fig. 5I and J). On the contrary, the luciferase activity was markedly reduced upon *ARID1A* overexpression (Fig. 5I and J). Collectively, our results suggest that ARID1A exhibits an inhibitory effect on cell survival and colony formation via transcriptional suppression of *c-MYC* and *PARP1*.

### KD of *ARID1A* stimulates endogenous DDR and activates the PI3K/AKT pathway

**Fig. 6.**
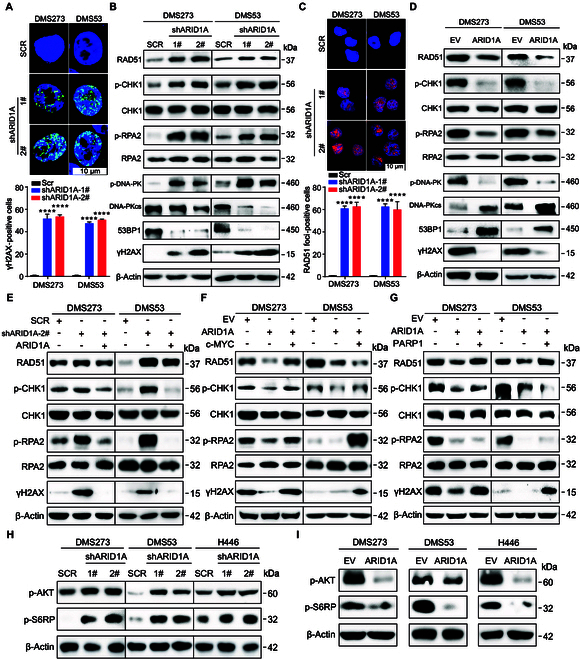
ARID1A participates in DDR in SCLC through the c-MYC/PARP axis. (A and B) Western blot analysis of DDR-related gene in DMS273 and DMS53 cells following *ARID1A* KD (A) or OE (B). (C and D) Representative images of immunofluorescence staining for γ-H2AX (C) and RAD51 (D) in DMS273 and DMS53 cells following *ARID1A* KD. Cells with more than 5 foci were considered positive. Quantification of γ-H2AX /RAD51 fluorescence intensities from 3 independent experiments was shown as mean ± SD. Statistical analysis was performed using 2-tailed unpaired Student’s *t* tests. ****P < 0.0001. (E to G) Western blot analysis of the indicated proteins following *ARID1A* (E), *c-MYC* (F), or *PARP1* (G) overexpression in *ARID1A* KD DMS273 and DMS53 cells. (H and I) Western blot analysis of PI3K downstream phospho-proteins in DMS273 and DMS53 cells following *ARID1A* KD (H) or OE (I).

To further explore the underlying mechanism that ARID1A exerts as a tumor suppressor in SCLC, we examined the effect of ARID1A on the DNA damage response (DDR). The immunofluorescence confirmed much more elevated γH2AX foci (a well-recognized marker for DSBs) in *ARID1A*-depleted cells than in *ARID1A*-proficient cells (Fig. 6A). Concomitantly, Western blot analysis showed that RAD51 expression and phosphorylation of CHK1 and RPA2 were robustly induced (Fig. 6B and Fig. S5A). Meanwhile, 53BP1 expression was suppressed (Fig. 6B and Fig. S5A). Immunofluorescence further confirmed elevated RAD51 foci (Fig. 6C) in *ARID1A*-depleted cells than in control cells. Similar results were also observed when *ARID1A* was transiently silenced by siRNA (Fig. S4A). In contrast, overexpression of *ARID1A* led to diminished expression of RAD51 and phosphorylation of CHK1, RPA2, and γH2AX, as well as increased 53BP1 expression in DMS273, DMS53, and H446 cells (Fig. 6D and Fig. S5A). Furthermore, ectopic expression of *ARID1A*, *c-MYC*, and PARP1 could reverse the activation of CHK1 and RPA2 and induction of γH2AX by *ARID1A* depletion (Fig. 6E to G). Taken together, these results suggest that ARID1A plays a pivotal role in modulating the DDR and stabilizing the genome.

A previous study reported that ARID1A activity was correlated with p-AKT signals in cancers [28,29], and the key components of the phosphatidylinositol 3-kinase (PI3K) pathway were often either mutated or dysregulated in SCLC (Fig. S6A and B). We thus measured the PI3K signaling activity following the KD/overexpression of *ARID1A*. Surprisingly, robustly elevated p-AKT and p-S6RP signals were observed following *ARID1A* silencing (Fig. 6H). However, this effect was inhibited by *ARID1A* overexpression (Fig. 6I), indicating that one of the mechanisms of ARID1A inhibition on cell survival in SCLC is through blockade of the PI3K/AKT signaling.

### *ARID1A* deficiency sensitizes SCLC cells to DNA damage inducers

**Fig. 7.**
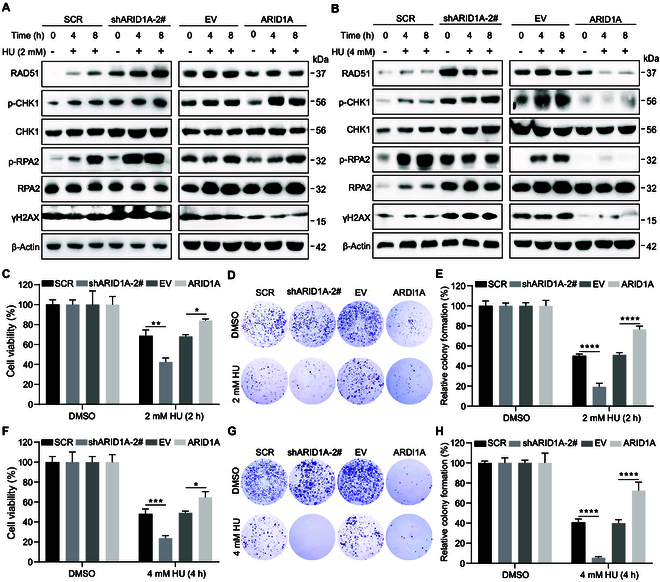
The effects of ARID1A on the response to replication stress and DSB induced by HU. (A and B) Western blot analysis of DDR-related genes following the exposure to 2 mM HU (A) and 4 mM HU (B) for the indicated times in *ARID1A* KD or OE DMS273 cells. (C to H) Cell viability (C and F) and clonal formation (D, E, G, and H) assays following 2 mM HU (C to E) and 4 mM HU (F to H) in *ARID1A* KD or OE DMS273 cells. *n* ≥ 3 independent experiments. Data are shown as the mean ± SEM; *n* ≥ 3 independent experiments. Statistical analysis was performed using a one-way ANOVA. *P < 0.05, **P < 0.01, ***P < 0.001, ****P < 0.0001.

To further address the effect of ARID1A on replication stress response (RSR) and DSB, we treated the cells with 2 mM hydroxyurea (HU) and detected the expression of DDR genes. Both *ARID1A*-defective and proficient cells exhibited promoted HU-induced p-RPA2, even though *ARID1A*-depleted cells have a much higher basal DDR activity than control cells, indicating induction of efficient DSB resection in both types of cells (Fig. 7A and Fig. S7A). Similarly, the levels of p-CHK1 and RAD51 were also remarkably induced by HU in both types of cells (Fig. 7A and Fig. S7A). On the contrary, in cell lines overexpressing *ARID1A*, the accumulation of p-CHK1 and p-RPA2 was retarded (Fig. 7A and Fig. S7B). These data suggest that ARID1A is not required to initiate RSR in response to external replication stress stimuli. After DSBs were induced by 4 mM HU, we found that in the *ARID1A* depletion group, although the level of p-CHK1 continued to be induced in a time-dependent manner, the levels of p-RPA2 and RAD51 were remarkably down-regulated (Fig. 7B and Fig. S7C). Once *ARID1A* was overexpressed in SCLC cells, the opposite patterns were observed (Fig. 7B and Fig. S7D). These results indicated that maintenance of the RSR signaling is compromised by *ARID1A* loss. To delineate the effect of *ARID1A* dysexpression on cell survival and clonogenicity after an external genotoxic insult, stable cell lines with *ARID1A* KD were exposed to 2 mM HU for 2 h and 4 mM HU for 4 h, and cell viability and clonogenic assays were carried out. The results demonstrated that *ARID1A* overexpression rendered cells more resistant to HU treatment (Fig. 7C to H). On the contrary, *ARID1A*-depleted cells were more sensitive to HU treatment than the control cells since there was much less cell survival and clonogenicity after *ARID1A* silencing (Fig. 7C to H). These results suggest that ARID1A can precisely respond to replication stress stimuli and DNA damage.

### *ARID1A* deficiency confers BET inhibitor sensitivity and BRD-K98645985 as a novel therapeutic drug in SCLC

**Fig. 8.**
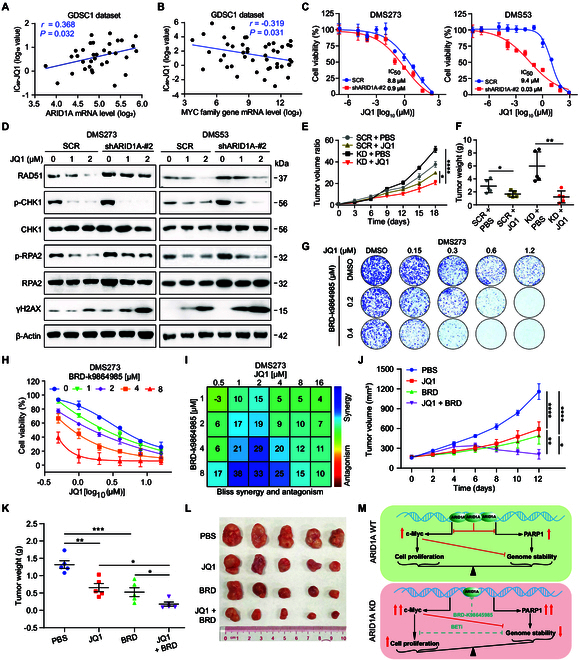
*ARID1A* deficiency enhances JQ1 sensitivity and identifies BRD-K98645985 as a novel therapeutic candidate in SCLC. (A and B) Pearson correlation analysis of IC_50_ values of JQ1 from the GDSC1 dataset and the mRNA level of *ARID1A* (A) and *MYC* family gene (B). (C) Dose–response analysis of JQ1 treatment on cell viability upon *ARID1A* KD DMS273 and DMS53 cells. (D) Western blot analysis of DDR-related genes following the exposure to JQ1 at indicated concentrations in *ARID1A* KD DMS273 and DMS53 cells. (E) Tumor volume curves of DMS273 control cells (SCR) and *ARID1A* KD cells upon exposure to JQ1 in vivo. KD denotes shARID1A-2#. (F) Scatterplot representing tumor weights of DMS273 control cells (SCR) and *ARID1A* KD cells upon exposure to JQ1 at the endpoint of experiments in vivo. KD refers to shARID1A-2#. (G) Colony formation assays demonstrating the effect of BRD-K98645985 on the cytotoxicity of JQ1 in DMS273 cells. (H) Drug–response curves of BRD-K98645985 and JQ1 combination in DMS273 cells. Cells were treated with escalating doses for 72 h, with viability measured by CellTiter-Glo. (I) Bliss analysis (Combenefit software) showing synergistic effects. (J) Tumor growth curves in xenograft mice treated with JQ1, BRD-K98645985 (BRD), or their combination (JQ1 + BRD). (K) Scatterplot depicting tumor weights from xenograft mice at the endpoints of the experiments. (L) Representative images of xenograft tumors. Data are mean ± SEM, and statistical analysis was performed using a one-way ANOVA. *P < 0.05, **P < 0.01, ***P < 0.001, ****P < 0.0001. (M) Proposed model depicting the function of ARID1A to modulate cell proliferation and genome stability through *c-MYC* and *PARP1*.

It has been found that targeting the DNA damage repair system is a promising strategy for treating SCLC. Our previous studies found that the BET bromodomain inhibitor (BETi) JQ1 can inhibit c-MYC signaling [30]. To address whether the deletion of *ARID1A* increases the sensitivity of SCLC cells to BETi, the correlation analysis of microarray data and IC_50_ (inhibitory concentration) values of JQ1 from the GDSC1 dataset was performed. Our analysis revealed that a positive correlation was observed between the IC_50_ values of JQ1 and *ARID1A* expression levels in SCLC, suggesting that cellular sensitivity to JQ1 treatment may be enhanced by *ARID1A* KD (Fig. 8A). Conversely, an inverse correlation was identified between JQ1 IC_50_ values and the expression levels of *MYC* family genes, indicating that increased JQ1 sensitivity is associated with elevated MYC expression (Fig. 8B). Moreover, through cell viability assay, we found that the sensitivity of *ARID1A*-defective cells to BETi JQ1 was significantly increased (Fig. 8C and Fig. S8A). Clonogenic assays further confirmed that *ARID1A* silencing rendered the sensitivity of SCLC cells to JQ1 (Fig. S8B and C). To gain further insight into the molecular mechanisms underlying sensitivity to JQ1 in *ARID1A*-deficient SCLC cells, we treated the cells with different concentrations of JQ1 for 24 hours and then assessed the expression of DDR genes by Western blot. Consistent with our previous works, JQ1 alone resulted in dose-dependent down-regulation of p-CHK1, p-RPA2, and RAD51 in DMS273 and DMS53 cells (Fig. 8D). The above data suggest that JQ1 confers tumor sensitivity by inhibiting the level of RSR and the ability of homologous recombination repair in *ARID1A*-defective cells.

To test the effect of *ARID1A* depletion on the activity of JQ1 in vivo, xenograft tumors with *ARID1A* KD were established by subcutaneous injection of DMS273 control and *ARID1A* KD cells and treated with JQ1. After 3 weeks of treatment, JQ1 alone retarded tumor growth in the presence of *ARID1A*. However, treatment with JQ1 inhibited tumor growth and reduced tumor weight much more in *ARID1A* KD tumors than in control tumors (Fig. 8E and F). Notably, no significant difference in weight loss and animal mortality was observed (Fig. S8D). These results show that JQ1 treatment and *ARID1A* depletion achieve a synthetic lethal effect by reducing RSR and cell repair capacity.

A recent study has identified a 12-membered macrolactam compound (BRD-K98645985, BRD) that selectively targets the ARID1A-containing BAF complex, effectively inhibiting its transcriptional repressive function while demonstrating minimal cytotoxicity [31]. Our investigation further revealed that BRD exhibits potent inhibitory effects on SCLC (Fig. S8E and F), significantly suppressing colony formation capacity (Fig. S8G). These findings suggest that BRD may serve as a promising therapeutic agent for SCLC through its specific targeting of ARID1A. Notably, combination therapy with BRD and JQ1 demonstrated superior therapeutic efficacy compared to JQ1 monotherapy (Fig. 8G and Fig. S8H), with Bliss independence analysis confirming a robust synergistic effect in vitro (Fig. 8H and I and Fig. S8I and J). These results highlight the potential of dual epigenetic targeting to overcome drug resistance and improve outcomes in SCLC, positioning BRD as a novel candidate for combination regimens in ARID1A-dependent malignancies.

In addition, we have further evaluated the therapeutic efficacy of BRD in combination with JQ1 in SCLC xenograft models. The results demonstrate that while both BRD and JQ1 monotherapies effectively inhibit tumor growth in vivo, their combination exhibits superior antitumor activity (Fig. 8J to L). Importantly, neither single-agent treatment nor the combination therapy showed significant adverse effects on normal mouse growth (Fig. S8K), confirming their favorable safety profile. These findings robustly support the therapeutic potential of targeting ARID1A in SCLC and significantly enhance the translational relevance of our study.

## Discussion

SCLC is a devastating neuroendocrine tumor with high mortality and recurrence rates [32]. Currently, chemotherapy combined with radiotherapy is still the mainstay of treatment for SCLC. However, most patients are diagnosed at an advanced stage due to the lack of effective biomarkers in early diagnosis and prognosis evaluation of SCLC [33]. Therefore, finding effective biomarkers and targets is urgently needed to improve the clinical outcome of patients with SCLC. Through bioinformatics analysis of RNA-seq data integrated with clinicopathological information, we revealed that SCLC patients with higher *ARID1A* expression had better PFS and OS than patients with low *ARID1A* expression. Furthermore, our functional experiments showed that the *ARID1A*-defective cells had enhanced cell survival and colony-forming abilities, suggesting a tumor-suppressive role of *ARID1A* in SCLC. Furthermore, we discovered that ARID1A exerted a tumor-suppressive function through transcriptional suppression of *c-MYC* and *PARP1*. The up-regulation of these 2 proteins activates both the RSR and DSB repair pathways, creating a therapeutic vulnerability that can be effectively targeted through combined inhibition using BET inhibitor JQ1 and BRD-K98645985-mediated ARID1A targeting.

ARID1A, a subunit of the SWI/SNF family, is a chromatin remodeling complex that functions through ATP hydrolysis and energy release and exhibits a synthetic lethal effect with the ATR/CHK1 pathway [22,24]. However, how it participates in maintaining genome integrity remains unexplored. In *ARID1A*-defective SCLC cells, p-CHK1 and p-RPA2 were significantly activated, indicating that *ARID1A* depletion leads to intracellular activation of the RSR and DSB repair signaling. However, the *ARID1A* depletion-induced activation of the RSR and DSB response did not confer a survival advantage to the cells. One possible explanation would be that ARID1A acts as a critical mediator to balance cell proliferation and genome integrity. ARID1A facilitates the binding of SWI/SNF complexes to the promoter regions of oncogenes and tumor suppressor genes, thereby altering the accessibility of chromatin and chromatin structure to specific transcription factors. In our case, ARID1A could recruit SWI/SNF complexes to the promoters of *c-MYC* and *PARP1,* suppressing their expression. On the one hand, ARID1A loss might change nucleosome space and allow transcription factors such as c-MYC to access the DNA sequence to activate its expression. c-MYC is a double-edged sword that has an activity to promote cell proliferation and genome instability [34]. Therefore, c-MYC up-regulation would elicit a strong RSR and trigger the DSB repair signaling [35]. On the other hand, PARP1, a well-characterized defender of genome integrity, is elevated upon ARID1A depletion. PARP1 elevation could counteract c-MYC-induced genome instability. ARID1A has been proposed to have the features of a gatekeeper and the features of a caretaker, but the underlying mechanism needs to be better understood. Herein, we propose that ARID1A possesses a dual role in cell proliferation and genome stability via suppression of *c-MYC* and *PARP1* (Fig. 8M).

Bromodomain and outdomain (BET) proteins, mainly composed of BRD2, BRD3, and BRD4 proteins, function as epigenetic readers and transcription coactivators and are currently recognized as therapeutic cancer targets [36,37]. Current studies have found that BET inhibitors have impressive effects in single-drug or combination therapy for various cancers, but the mechanisms of action are different. A recent study found an increase of 6,197 BRD4-binding sites in *ARID1A* knockout breast cancer cells, indicating a significant increase in BRD4 chromatin binding activity [10]. Our data demonstrated that *ARID1A*-defective SCLC cells are susceptible to BETis. The increased sensitivity might be due to the activation of the Myc signaling, which enhances BRD4 activity to read chromatin modifications. So, our results are in line with the observation in breast cancer cells after *ARID1A* KD. However, the underlying mechanisms merit further investigation. Our investigation also suggests that *ARID1A*-depleted SCLC cells show superior sensitivity to BET inhibitors. JQ1 displayed the highest cytotoxic activity. JQ1 treatment could strongly inhibit the ATR-CHK1 and the DSB repair signaling, as both p-CHK1 and p-RPA2 signals were remarkably blocked by JQ1.

In conclusion, our experiment reported for the first time that ARID1A functions as a tumor suppressor gene in SCLC cells, and *ARID1A* expression could be informative for SCLC patient prognosis in terms of survival. Furthermore, *ARID1A* depletion activates the response to RSR and DSB by activating the expression of c-MYC and PARP1. In addition, our data further demonstrate that *ARID1A* depletion enhances the sensitivity of SCLC cell lines to the BET inhibitor JQ1, and the compound BRD-K98645985 exerts antitumor effects against SCLC through targeted inhibition of ARID1A while also synergizing with JQ1 to suppress SCLC growth. In conclusion, our experiment provides a new direction for SCLC’s diagnosis, prognosis, and treatment.

## Materials and Methods

### Cell culture

Human lung cell lines were maintained in RPMI 1640 medium supplemented with 10% fetal bovine serum (FBS) and 1% penicillin/streptomycin (P/S), and 293T cells were cultured in Dulbecco’s modified essential medium (DMEM) containing 10% FBS and 1% P/S. All cells were cultivated in a humidified incubator at 37 °C with 5% CO_2_. A549, PC9, DMS273, H446, SHP77, DMS53, H69, H82, H526, and DMS79 cell lines were used in this study. RPMI 1640 and DMEM medium, penicillin, and streptomycin were purchased from Gibco, Life Technologies (Carlsbad, CA, USA). All cells have been routinely tested for pathogens to prevent Mycoplasma contamination.

### Antibodies and drugs

Primary antibodies against the following proteins were used for immunoblotting: ARID1A [Cell Signaling Technology (CST), #12354S, 1:1,000], c-MYC (CST, #5605, 1:1,000), PARP1 (Active Motif, #39559,1:5,000), phospho-Akt (Ser^473^) (CST, #9271, 1:1,000), phospho-S6 ribosomal protein (Ser^235/236^) (CST, #4858, 1:1,000), phospho-CHK1 (Ser^317^) (CST, #12302, 1:1,000), p-RPA2/32 (Ser^4^/Ser^8^) (Novus, NB100-544, 1:5,000), γ-H2AX (CST, #9718, 1:1,000), RAD51 (Abcam, ab133534, 1:1,000), RPA2/32 (Abcam, ab2175, 1:1,000), CHK1 (CST, 2360S, 1:1,000), β-actin (Transgen, HC201-02, 1:10,000), DNA-PKcs (CST, #4602, 1:1,000), p-DNA-PK (CST, #68716, 1:1,000), and 53BP1 (CST, #4937, 1:1,000). The following antibodies are used for ChIP and immunoprecipitation (IP) experiments: acetyl-histone H3 (Lys^9^) (CST, #9649), ARID1A (CST, #12354S), c-MYC (Abcam, ab152146), PARP1 (Active Motif, 39559), acetyl-H3K27 (Abcam, ab177178), and trimethyl H3K27 (Abcam, ab6002). The following antibodies are used for IHC analysis: c-MYC (CST, #5605), PARP1 (Active Motif, #39559), ARID1A (Abcam, ab272905), Ki67 (CST, #9027), RAD51 (Abcam, ab133534), and γH2AX (CST, #9718). All the antibodies were diluted with 5% bovine serum albumin (BSA) and stored at 4 °C. JQ1 and BRD-K98645985 were obtained from Selleck Chemical (Shanghai, China). All drugs were dissolved in dimethyl sulfoxide (DMSO) (Sigma-Aldrich, Saint Louis, MO, USA) to obtain concentrated stock solutions and used at the concentrations described.

### SCLC dataset analysis

RNA-seq data from 50 SCLC and 75 LUAD cell lines, and general information for these cell lines, were downloaded from https://depmap.org/portal/download/. Expression data of 81 and 69 human primary SCLC tissues and 7 nontumor lung tissues, and corresponding clinical information were downloaded from George et al. (2015) and the GSE60052 dataset, respectively. Microarray data of 18 primary human SCLC and adjacent noncancerous tissues were obtained from GSE149507. The transcriptomic profiles of 107 SCLC cases along with their paired normal tissues adjacent to the tumors were acquired from the National Genomics Data Center’s Genome Sequence Archive repository (GSA-Human accession HRA003419, accessible via https://bigd.big.ac.cn/gsa-human). In parallel, comprehensive protein expression profiles and corresponding clinical pathology details from 112 SCLC subjects with matched nonmalignant control samples were retrieved through the OMIX biomedical data repository (reference code OMIX002489) maintained by the Beijing Institute of Genomics under the Chinese Academy of Sciences’ bioinformatics network platform (https://ngdc.cncb.ac.cn/omix). The GDSC dataset was obtained from https://www.cancerrxgene.org/downloads/anova. Expression data for *ARID1A* and *MYC* genes and the IC_50_ values of JQ1 were extracted from the above datasets. The Pearson correlation coefficient was used to analyze the correlations between the mRNA level of *ARID1A* or *MYC* and the IC_50_ value of JQ1. The mutation profiles of *ARID1s* were analyzed by a standard processing pipeline in the cBioPortal (https://www.cbioportal.org). The SCLC dataset that contained 249 pathological reports was chosen for comprehensive genomic analysis.

### Cell viability and clonogenic assays

Cells seeded in 96-well plates at a density of 3,000 cells per well were treated with small compounds as indicated. After incubation for 72 h at 37 °C, 20 μl of CellTiter-Glo (CTG) solution was added to each well and incubated in the dark for 10 min at room temperature. The fluorescence intensity was recorded on an Envision PerkinElmer porous plate microplate reader (PerkinElmer, Waltham, MA, USA). Cells in each group were tested with at least 3 independent biological replicates. For the clonogenic assay, cells were plated at a density of 1,000 to 3,000 cells per well in 6-well plates. After 24 h, the media were replaced with complete media containing various dilutions of the drugs and cultured for 14 to 21 d in a humidified incubator of 5% CO_2_ at 37 °C. The media were changed every 3 to 4 d. Colonies were fixed with methanol and stained with 2% crystal violet for 20 min. For colorimetric assays, the stained colonies were quantified by ImageJ.

### Transfections with siRNA and plasmid DNA

For siRNA-mediated *ARID1A*, *c-MYC*, and *PARP1* KD experiments, the siRNA was purchased from General Biosystem Co. Ltd. (Hefei, China). siRNA sequences were listed in Table S1. Cells were seeded into 6-well plates at 60% density and transfected with siRNA or plasmid DNA of interest using an Effectene transfection reagent according to the manufacturer’s instructions. Cells were collected at 48 h post-transfection for Western blot and RT-qPCR analysis and at 72 h for cell viability assay.

### EdU assay

Cells were treated with 5-ethynyl-2’-deoxyuridine (EdU) for 2 h at 37 °C under the prescribed conditions at a final concentration of 10 μM. After being washed with phosphate-buffered saline (PBS) twice, the cells were fixed with 4% paraformaldehyde at room temperature for 15 min and permeated with 0.3% Triton X-100 for 15 min. Cells were then washed with PBS containing 3% BSA 3 times, followed by the detection of EdU signals using a Leica confocal microscope.

### Virus package and construction of stable cell lines

The short hairpin RNAs (shRNAs) were designed using the AsiDesigner software (https://rnaidesigner.thermofisher.com/rnaiexpress/sort.do) and subcloned into the pLKO.1-puro lentiviral vector. The shRNA sequences used in this study are summarized in Table S2. Lentiviral particles expressing shRNAs were produced in 293T cells according to the manufacturer’s instructions. Briefly, 293T cells were transfected with psPAX2 (#12260, AddGene, MA, USA), pMD2.G plasmid (#12259, AddGene), and pLKO.1 plasmid containing nontargeting shRNA (SCR) or ARID1A shRNAs (sh*ARID1A*) or pLenti-puro-ARID1A (#39478, AddGene). Viral particles were collected after 48 and 72 h post-transfection. Cells were infected with lentiviral particles containing 8 μg/ml polybrene (Sigma-Aldrich, Steinheim, Germany). Puromycin (Solarbio, Beijing, China) at 2 μg/ml was added to the medium for selection at 48 h post-infection. For induction of *ARID1A*, cells were harvested at 48 h post-incubation with Dox (1 μg/ml) for Western blot and RT-qPCR analysis, 72 h for cell viability assay, and 14 d for colony formation assay. The ectopic expression of *ARID1A* was verified by Western blotting at 48 h post-induction.

### Drug treatment

For drug treatment studies, SCLC cells were treated with 2 mM HU for 4 or 8 h to induce replication stress or with 4 mM HU to generate DSBs. For JQ1 treatment, cells were exposed to 1 or 2 μM JQ1 (DMSO as vehicle control) for 24 h before harvest. Long-term clonogenic assays employed specified concentrations of JQ1 and BRD-K98645985 for 7 to 10 d. Drug synergy was assessed by 72 h cotreatment with JQ1 and BRD-K98645985, followed by CellTiter-Glo viability assays, with synergy indices calculated using Combenefit software (CRUK Cambridge Institute).

### RT-qPCR and ChIP-PCR

The Trizol RNA extraction kit was used to extract the total RNA from the cells. Next, total RNA (1 μg) was reverse-transcribed to cDNA using the Novizam reverse transcription kit with a genomic DNA (gDNA) eraser according to the manufacturer’s instructions. Finally, qPCR detection and analysis were performed using the Novizam fluorescence quantitative kit on a Roche LightCycler 96 Real-Time PCR System. The relative expression levels of the tested genes were normalized to the housekeeping gene β-actin. The primer sequences used for qPCR are summarized in Table S3. For ChIP-PCR analysis, the cellular gDNA was decomposed and lysed by ultrasound, then treated with RNA enzyme and protease, and rotated overnight with ARID1A antibody at 4 °C. DNA was purified from the reverse cross-linked chromatin using a PCR purification kit on the next day and detected by qPCR. The primers used for ChIP-qPCR were derived from the literature [38] and summarized in Table S4.

### Cell apoptosis assays

To detect apoptosis, the cells were treated under specified conditions for 48 h and then incubated with annexin V-fluorescein isothiocyanate and propidium iodide for annexin detection. Cell apoptosis was detected using FACSCalibur (Cytexpert, Beckman Coulter, Brea, CA, USA), and data were analyzed using FlowJo V10 software (Flowjo LLC, Ashland, Oregon, USA).

### Immunofluorescence staining

For indirect immunofluorescence, cells were washed with PBS and fixed with 4% paraformaldehyde for 5 min, and then permeabilized with 0.5% Triton X-100 for 10 min. After blocking with 5% BSA for 30 min, the primary antibodies were applied overnight at 4 °C. On the second day, a fluorescent secondary antibody was applied in the shade at room temperature for 1 h. After elution, slides were sealed with an anti-quenching agent containing 4′,6-diamidino-2-phenylindole (DAPI) for further image acquisition using a Leica confocal microscope.

### Dual-luciferase reporter assay

The promoter sequences of *c-MYC* and *PARP1* were subcloned into the pGL4 vector by a homologous recombination method. Then, the vector was cotransfected with pGL4, pRL-TK, pLKO-sh*ARID1A*, or pLenti-puro-ARID1A into 293T cells using Effectene transfection reagent. Luciferase activities were detected using the Novizan double luciferase detection kit (Vazyme, DD1205-01) 48 h after transfection, with Renilla luciferase activity as the internal control.

### Mouse model of xenograft

A 100-μl suspension of 5 × 10^6^ ARID1A KD, *ARID1A* overexpressed (OE), and control DMS273 and H446 cells in an equal volume of Matrigel (BD Biosciences, Franklin, NJ, USA) was injected subcutaneously in the dorsal flank of 4-week-old female nonobese diabetic (NOD)/severe combined immunodeficient (SCID) mice (Beijing Vitong Lihua Laboratory Animal Co. Ltd.). To induce ARID1A expression in tumors, after the subcutaneous tumors reached 4 to 5 mm in diameter, mice were fed with drinking water (sucrose: 20 g/l) containing Dox (2 g/l) for 15 d. For JQ1 monotherapy experiments, treatment was initiated when the mean tumor volume reached approximately 100 mm^3^, with intraperitoneal administration of either PBS (vehicle control) or JQ1 (30 mg/kg) every other day (Q2D). In experiments evaluating JQ1 and BRD-K98645985 as single agents or in combination, JQ1 was administered at the same dose (30 mg/kg, intraperitoneally, Q2D), while BRD-K98645985 was dosed at 20 mg/kg (intraperitoneally, Q2D). For combination therapy, both agents were delivered concurrently following the same Q2D schedule. The tumor volume and body weight of the mice were measured every 2 to 3 d. Tumor sizes were measured using a caliper, and tumor volumes were determined using the following equation: tumor volume [mm^3^] = (tumor length × tumor width^2^)/2. After reaching the endpoint, the mice were euthanized, and tumors were harvested for H&E staining and IHC analysis. All mouse experiments were carried out according to the protocol approved by the Hefei Institute of Physical Science Animal Care and Use Committee.

### Histological and IHC analyses

After fixation with 4% paraformaldehyde in PBS overnight, the tumor tissues were embedded in paraffin and cut at 4 μm. The slides were stained with hematoxylin and eosin (H&E). IHC studies were performed on formalin-fixed, paraffin-embedded sections. After retrieval using a universal retrieval buffer, the slides were incubated with primary antibodies at 4 °C overnight. The next day, slides were rinsed and incubated with the respective secondary antibody. Images were photographed using a Leica confocal microscope. The antibodies used in IHC staining were as follows: Ki67 (CST, 9449, 1:300), ARID1A (CST, #12354S, 1:1,000), c-MYC (CST, #5605, 1:300), PARP1 (Active Motif, #39559, 1:500), RAD51 (Abcam, ab133534, 1:200), and γH2AX (Ser^139^, CST 2577, 1:500). Three to 5 random fields at 40× magnification were collected for each tumor sample.

### Quantification and statistical analysis

The band intensities from the Western blot were quantified by gray value by area using ImageJ software. To quantify the IHC staining of SCLC specimens, the signals were quantified by integrated optical density (IOD), equal to optical density by area, using Image Pro Plus software. For the prognostic significance study, patients were classified into the *ARID1A* low and *ARID1A* high groups based on median expression levels of *ARID1A* in the cohort of patients with SCLC. OS and PFS curves were calculated with the Kaplan–Meier product-limit method and were analyzed with the log-rank test. Data were listed as mean ± SD or SEM of at least 3 independent experiments. The quantitative results were analyzed using the GraphPad Prism 6.0 software with a 2-tailed unpaired *t* test or one-way analysis of variance (ANOVA). *P* < 0.05 was considered statistically significant. *P < 0.05, **P < 0.01, ***P < 0.001, ****P < 0.0001.

## Data Availability

All data generated or analyzed during this study are included in this published article.

## References

[1] Oronsky B, Reid TR, Oronsky A, Carter CA. What’s new in SCLC? A review. Neoplasia. 2017;19(10):842–847. 28888101 10.1016/j.neo.2017.07.007 PMC5596356

[2] Gay CM, Stewart CA, Park EM, Diao L, Groves SM, Heeke S, Nabet BY, Fujimoto J, Solis LM, Lu W, et al. Patterns of transcription factor programs and immune pathway activation define four major subtypes of SCLC with distinct therapeutic vulnerabilities. Cancer Cell. 2021;39(3):346–360.e347. 33482121 10.1016/j.ccell.2020.12.014 PMC8143037

[3] Hayashi R, Inomata M. Small cell lung cancer; recent advances of its biology and therapeutic perspective. Respir Investig. 2022;60(2):197–204. 10.1016/j.resinv.2021.10.008 34896039

[4] Wu JN, Roberts CW. ARID1A mutations in cancer: Another epigenetic tumor suppressor? Cancer Discov. 2013;3(1):35–43. 23208470 10.1158/2159-8290.CD-12-0361 PMC3546152

[5] Wilson BG, Roberts CW. SWI/SNF nucleosome remodellers and cancer. Nat Rev Cancer. 2011;11(7):481–492. 21654818 10.1038/nrc3068

[6] Hargreaves DC, Crabtree GR. ATP-dependent chromatin remodeling: Genetics, genomics and mechanisms. Cell Res. 2011;21(3):396–420. 21358755 10.1038/cr.2011.32 PMC3110148

[7] Lo YH, Kolahi KS, du Y, Chang CY, Krokhotin A, Nair A, Sobba WD, Karlsson K, Jones SJ, Longacre TA, et al. A CRISPR/Cas9-engineered ARID1A-deficient human gastric cancer organoid model reveals essential and nonessential modes of oncogenic transformation. Cancer Discov. 2021;11(6):1562–1581. 33451982 10.1158/2159-8290.CD-20-1109 PMC8346515

[8] Wang K, Kan J, Yuen ST, Shi ST, Chu KM, Law S, Chan TL, Kan Z, Chan ASY, Tsui WY, et al. Exome sequencing identifies frequent mutation of ARID1A in molecular subtypes of gastric cancer. Nat Genet. 2011;43(12):1219–1223. 22037554 10.1038/ng.982

[9] Dong X, Song S, Li Y, Fan Y, Wang L, Wang R, Huo L, Scott A, Xu Y, Pizzi MP, et al. Loss of ARID1A activates mTOR signaling and SOX9 in gastric adenocarcinoma—Rationale for targeting ARID1A deficiency. Gut. 2022;71(3):467–478. 33785559 10.1136/gutjnl-2020-322660 PMC9724309

[10] Nagarajan S, Rao SV, Sutton J, Cheeseman D, Dunn S, Papachristou EK, Prada JG, Couturier D-L, Kumar S, Kishore K, et al. ARID1A influences HDAC1/BRD4 activity, intrinsic proliferative capacity and breast cancer treatment response. Nat Genet. 2020;52(2):187–197. 31913353 10.1038/s41588-019-0541-5 PMC7116647

[11] Shen J, Peng Y, Wei L, Zhang W, Yang L, Lan L, Kapoor P, Ju Z, Mo Q, Shih IM, et al. ARID1A deficiency impairs the DNA damage checkpoint and sensitizes cells to PARP inhibitors. Cancer Discov. 2015;5(7):752–767. 26069190 10.1158/2159-8290.CD-14-0849 PMC4497871

[12] Gupta S, Albertson DJ, Parnell TJ, Butterfield A, Weston A, Pappas LM, Dalley B, O’Shea JM, Lowrance WT, Cairns BR, et al. Histone deacetylase inhibition has targeted clinical benefit in ARID1A-mutated advanced urothelial carcinoma. Mol Cancer Ther. 2019;18(1):185–195. 30301863 10.1158/1535-7163.MCT-17-0957 PMC6350908

[13] Samartzis EP, Samartzis N, Noske A, Fedier A, Caduff R, Dedes KJ, Fink D, Imesch P. Loss of ARID1A/BAF250a-expression in endometriosis: A biomarker for risk of carcinogenic transformation? Mod Pathol. 2012;25(6):885–892. 22301703 10.1038/modpathol.2011.217

[14] Sun X, Wang SC, Wei Y, Luo X, Jia Y, Li L, Gopal P, Zhu M, Nassour I, Chuang JC, et al. Arid1a has context-dependent oncogenic and tumor suppressor functions in liver cancer. Cancer Cell. 2018;33(5):151–152. 10.1016/j.ccell.2017.12.011 PMC5783571 29316428

[15] Wu C, Lyu J, Yang EJ, Liu Y, Zhang B, Shim JS. Targeting AURKA-CDC25C axis to induce synthetic lethality in ARID1A-deficient colorectal cancer cells. Nat Commun. 2018;9:3212. 30097580 10.1038/s41467-018-05694-4 PMC6086874

[16] Zhai Y, Kuick R, Tipton C, Wu R, Sessine M, Wang Z, Baker SJ, Fearon ER, Cho KR. Arid1a inactivation in an Apc- and Pten-defective mouse ovarian cancer model enhances epithelial differentiation and prolongs survival. J Pathol. 2015;238(1):21–30. 26279473 10.1002/path.4599 PMC4715504

[17] Wilson MR, Reske JJ, Holladay J, Neupane S, Ngo J, Cuthrell N, Wegener M, Rhodes M, Adams M, Sheridan R, et al. ARID1A mutations promote P300-dependent endometrial invasion through super-enhancer hyperacetylation. Cell Rep. 2020;33(6): Article 108366. 33176148 10.1016/j.celrep.2020.108366 PMC7682620

[18] Bui CB, le HK, Vu DM, Truong KDD, Nguyen NM, Ho MAN, Truong DQ. ARID1A-SIN3A drives retinoic acid-induced neuroblastoma differentiation by transcriptional repression of TERT. Mol Carcinog. 2019;58(11):1998–2007. 31365169 10.1002/mc.23091

[19] Rahmanto YS, Jung JG, Wu RC, Kobayashi Y, Heaphy CM, Meeker AK, Wang TL, Shih IM. Inactivating ARID1A tumor suppressor enhances TERT transcription and maintains telomere length in cancer cells. J Biol Chem. 2016;291(18):9690–9699. 26953344 10.1074/jbc.M115.707612 PMC4850306

[20] Watanabe R, Ui A, Kanno SI, Ogiwara H, Nagase T, Kohno T, Yasui A. SWI/SNF factors required for cellular resistance to DNA damage include ARID1A and ARID1B and show interdependent protein stability. Cancer Res. 2014;74(9):2465–2475. 24788099 10.1158/0008-5472.CAN-13-3608

[21] Loe AKH, Francis R, Seo J, du L, Wang Y, Kim JE, Hakim SW, Kim JE, He HH, Guo H, et al. Uncovering the dosage-dependent roles of Arid1a in gastric tumorigenesis for combinatorial drug therapy. J Exp Med. 2021;218(6): Article e20200219. 33822841 10.1084/jem.20200219 PMC8034383

[22] Mathur R. ARID1A loss in cancer: Towards a mechanistic understanding. Pharmacol Ther. 2018;190:15–23. 29730444 10.1016/j.pharmthera.2018.05.001

[23] Kurz L, Miklyaeva A, Skowron MA, Overbeck N, Poschmann G, Becker T, Eul K, Kurz T, Schönberger S, Calaminus G, et al. ARID1A regulates transcription and the epigenetic landscape via POLE and DMAP1 while ARID1A deficiency or pharmacological inhibition sensitizes germ cell tumor cells to ATR inhibition. Cancer. 2020;12(4):905. 10.3390/cancers12040905 PMC7226530 32272809

[24] Williamson CT, Miller R, Pemberton HN, Jones SE, Campbell J, Konde A, Badham N, Rafiq R, Brough R, Gulati A, et al. ATR inhibitors as a synthetic lethal therapy for tumours deficient in ARID1A. Nat Commun. 2016;7:13837. 27958275 10.1038/ncomms13837 PMC5159945

[25] Bitler BG, Aird KM, Garipov A, Li H, Amatangelo M, Kossenkov AV, Schultz DC, Liu Q, Shih IM, Conejo-Garcia JR, et al. Synthetic lethality by targeting EZH2 methyltransferase activity in ARID1A-mutated cancers. Nat Med. 2015;21(3):231–238. 25686104 10.1038/nm.3799 PMC4352133

[26] Bitler BG, Wu S, Park PH, Hai Y, Aird KM, Wang Y, Zhai Y, Kossenkov AV, Vara-Ailor A, Rauscher III FJ, et al. ARID1A-mutated ovarian cancers depend on HDAC6 activity. Nat Cell Biol. 2017;19(8):962–973. 28737768 10.1038/ncb3582 PMC5541905

[27] Liu F, Ying J, Yang K, Xiong X, Yang N, Wang S, Zhao W, Zhu H, Yu M, Wu J, et al. Deciphering the regulatory mechanisms and biological implications of ARID1A missense mutations in cancer. Cell Rep. 2024;43(11):114916. 39475510 10.1016/j.celrep.2024.114916

[28] Yang Y, Wang X, Yang J, Duan J, Wu Z, Yang F, Zhang X, Xiao S. Loss of ARID1A promotes proliferation, migration and invasion via the Akt signaling pathway in NPC. Cancer Manag Res. 2019;11:4931–4946. 31213911 10.2147/CMAR.S207329 PMC6549766

[29] Berns K, Sonnenblick A, Gennissen A, Brohée S, Hijmans EM, Evers B, Fumagalli D, Desmedt C, Loibl S, Denkert C, et al. Loss of ARID1A activates ANXA1, which serves as a predictive biomarker for trastuzumab resistance. Clin Cancer Res. 2016;22(21):5238–5248. 27172896 10.1158/1078-0432.CCR-15-2996

[30] Bian X, Wang X, Zhang Q, Ma L, Cao G, Xu A, Han J, Huang J, Lin W. The MYC paralog-PARP1 Axis as a potential therapeutic target in MYC paralog-activated small cell lung cancer. Front Oncol. 2020;10: Article 565820. 33134168 10.3389/fonc.2020.565820 PMC7578565

[31] Marian CA, Stoszko M, Wang L, Leighty MW, de Crignis E, Maschinot CA, Gatchalian J, Carter BC, Chowdhury B, Hargreaves DC, et al. Small molecule targeting of specific BAF (mSWI/SNF) complexes for HIV latency reversal. Cell Chem Biol. 2018;25(12):1443–1455.e1414. 30197195 10.1016/j.chembiol.2018.08.004 PMC6404985

[32] Zhang Z, Wu X, Bao S, Sun X, Yang F, Zhang Y, Yang Z, Zhang L, Chen R, Xing P, et al. Proteogenomic characterization of high-grade lung neuroendocrine carcinoma deciphers molecular diversity and potential biomarkers of different histological subtypes in Chinese population. Research. 2025;8: Article 0671. 40230612 10.34133/research.0671 PMC11994885

[33] Gazdar AF, Bunn PA, Minna JD. Small-cell lung cancer: What we know, what we need to know and the path forward. Nat Rev Cancer. 2017;17(12):725–737. 29077690 10.1038/nrc.2017.87

[34] Zhang H, Zhai X, Liu Y, Xia Z, Xia T, du G, Zhou H, Franziska Strohmer D, Bazhin AV, Li Z, et al. NOP2-mediated m5C modification of c-Myc in an EIF3A-dependent manner to reprogram glucose metabolism and promote hepatocellular carcinoma progression. Research. 2023;6: Article 0184. 37398932 10.34133/research.0184 PMC10313139

[35] Wang J, Shao F, Yu QX, Ye L, Wusiman D, Wu R, Tuo Z, Wang Z, Li D, Cho WC, et al. The common hallmarks and interconnected pathways of aging, circadian rhythms, and cancer: Implications for therapeutic strategies. Research. 2025;8: Article 0612. 40046513 10.34133/research.0612 PMC11880593

[36] Stathis A, Bertoni F. BET proteins as targets for anticancer treatment. Cancer Discov. 2018;8(1):24–36. 29263030 10.1158/2159-8290.CD-17-0605

[37] Belkina AC, Denis GV. BET domain co-regulators in obesity, inflammation and cancer. Nat Rev Cancer. 2012;12(7):465–477. 22722403 10.1038/nrc3256 PMC3934568

[38] Zhang W, Liu B, Wu W, Li L, Broom BM, Basourakos SP, Korentzelos D, Luan Y, Wang J, Yang G, et al. Targeting the MYCN–PARP–DNA damage response pathway in neuroendocrine prostate cancer. Clin Cancer Res. 2018;24:696–707. 29138344 10.1158/1078-0432.CCR-17-1872 PMC5823274

