# Supplementary material for: ARID1A Governs Genomic Stability and Proliferation in SCLC via c-MYC/PARP1 Suppression Driving Vulnerability to BET Inhibitors
Source: Research (Wash D C). 2025 Oct 2;8:0908. doi: 10.34133/research.0908 (PMC12489181; doi:10.34133/research.0908)
Supplement: Supplementary 1 — Figs. S1 to S8 Tables S1 to S4 [file research.0908.f1.zip › Supplementary information (revision).docx]

**ARID1A governs genomic stability and proliferation in SCLC via c-MYC/PARP1 suppression driving vulnerability to BET inhibitors**

Guozhen Cao^1, 2, 3, #^, Liying Ma^1, 2, 3, #^, Xueqin Dai^4, 5, #^, Peng Hou^1, 2, 3, #^, Xinhuang Yao^1^, Gongfeng Li^1, 2, 3^, Jiahui Zhang^1, 2, 3^, Ceshi Chen ^4, 5,^ *, Wenchu Lin^1, 3,^ *

^1^ The Second Affiliated Hospital, School of Medicine, The Chinese University of Hong Kong, Shenzhen & Longgang District People’s Hospital of Shenzhen, Shenzhen 518172, P. R. China

^2^ University of Science and Technology of China, Hefei 230036, Anhui, P. R. China

^3^ High Magnetic Field Laboratory, Chinese Academy of Sciences, Hefei 230031, Anhui, P. R. China

^4^ Yunnan Key Laboratory of Breast Cancer Precision Medicine, Yunnan Cancer Hospital, The Third Affiliated Hospital of Kunming Medical University, Peking University Cancer Hospital Yunnan, Kunming 650118, Yunnan, P. R. China

^5^ Yunnan Key Laboratory of Breast Cancer Precision Medicine, Academy of Biomedical Engineering, Kunming Medical University, Kunming 650000, Yunnan, P. R. China

**^#^ Denotes co-first authorship**

***Corresponding Author:**

Correspondence to:

Wenchu Lin, E-mail: [linwenchu@ustc.edu.cn](mailto:linwenchu@ustc.edu.cn;);

Ceshi Chen, E-mail: chenc@kmmu.edu.cn.

**Running title**: ARID1A Regulates SCLC via c-MYC/PARP1

**Supplementary Figure legends**

**Supplementary Figure 1**. **A**. Oncoprint of *ARID1A* and *ARID1B* mutations in 50 SCLC cell lines. **B**. Scatter plots of *ARID1A* expression in SCLC cell lines relative to lung adenocarcinoma (LUAD) cell lines from the GDSC-microarray dataset. Statistical analysis was performed using two-tailed unpaired Student’s *t*-tests for (**B**).

**Supplementary Figure 2.** The effect of ARID1A on apoptosis in SCLC cells. **A**. Western blot analysis of ARID1A expression following *ARID1A* knockdown (KD) and overexpression (OE) in H446 cells. **B**-**E**. Flow cytometry analysis of apoptosis in DMS273 and DMS53 cells following *ARID1A* KD (**B**) or OE (**D**). Panels (**C**) and (**E**) show the corresponding quantitative analysis for (**B**) and (**D**), respectively. Data are shown as the mean ± S.E.M.; *n* = 3 independent experiments. Statistical analysis was performed using two-tailed unpaired Student’s *t*-tests. ns, no significance.

**Supplementary Figure 3.** **A**, **B**. Body weight curves over time in DMS273 xenograft models with *ARID1A* knockdown (**A**) or overexpression (**B**). **C**. Tumor growth curves of H446 control (SCR or EV) versus *ARID1A*-depleted or overexpressing cells in xenograft models. **D**. Scatter plot of final tumor weights from each experimental group. **E**. Representative tumor images from all groups. **F**. Body weight trends of mice throughout the experimental period. Data are shown as the mean ± S.E.M.; Statistical analysis was performed using a one-way ANOVA. ****P* < 0.001, *****P* < 0.0001.

**Supplementary Figure 4.** ARID1A participates in the RSR and DSB repair signaling by regulating *c-MYC*/*PARP1* expression. **A**, **B**. Western blot analysis of the indicated proteins in DMS273, DMS53, and H446 cells treated with siRNAs against *ARID1A* (**A**) and in H446 cells following *ARID1A* knockdown or overexpression (**B**). **C**. Analysis of the correlation between *ARID1A* and *c-MYC* mRNA levels in 50 SCLC cell lines using data from the CCLE dataset.

**Supplementary Figure 5. A**. Western blot analysis of the corresponding proteins in H446 cells with *ARID1A* knockdown or overexpression.

**Supplementary Figure 6. A**, **B**. Oncoprint of genes encoding the key components of the PI3K/AKT signaling pathway in human primary SCLC (**A**) and SCLC cell lines (**B**).

**Supplementary Figure 7.** Protein quantification is shown in Figure 7, with β-actin as the normalization standard. **A**-**D**, Quantification of indicated protein intensities from three independent experiments with HU (2 mM) (**A**, **B**) and HU (4 mM) (**C**, **D**) in *ARID1A* knockdown (**A**, **C**) and overexpression (**B**, **D**). Data are shown as the mean ± S.E.M.; *n* = 3 independent experiments. Statistical analysis was performed using two-tailed unpaired Student’s *t*-tests. ns, no significance; **P* < 0.05, ***P* < 0.01, ****P* < 0.001, *****P* < 0.0001.

**Supplementary Figure 8.** *ARID1A* deficiency enhances JQ1 sensitivity and identifies BRD-K98645985 as a novel therapeutic candidate in SCLC. **A**. Analysis of cell viability in *ARID1A*-knockdown DMS273 and DMS53 cells following different time JQ1 treatment. **B**. Clonogenic survival assays of *ARID1A*-knockdown (KD) DMS273 and DMS53 cells treated with increasing concentrations of JQ1. **C**. Quantitative analysis of data shown in panel (**B**). KD denotes shARID1A-2#. **D**. Body weight curves of xenograft mice during treatment with JQ1 *in vivo*. KD denotes shARID1A-2#. **E**, **F**. Dose-response curves of BRD-K98645985 in DMS273 (**E**) and DMS53 (**F**) cells after 72 h (CellTiter-Glo). IC_50_ values were derived from the sigmoidal dose-response curves. **G**. Clonogenic assays illustrating the effect of BRD-K98645985 on DMS273 and DMS53 cells. **H**. Colony formation assays demonstrating the effect of BRD-K98645985 on the cytotoxicity of JQ1 in DMS53 cells. **I**. Drug-response curves of combining BRD-K98645985 and JQ1 in DMS53 cells. **J**. Bliss synergy calculation (Combenefit software) showing the synergistic effect of combining BRD-K98645985 and JQ1 in DMS53 cells. **K**. Body weight trends of xenograft mice treated with JQ1, BRD-K98645985 (BRD), or their combination (JQ1 + BRD) over time. Data are shown as the mean ± S.E.M.; *n* = 3 independent experiments. Statistical analysis was performed using two-tailed unpaired Student’s *t*-tests. ***P* < 0.01, ****P* < 0.001.
