# Supplementary material for: ARID1A Governs Genomic Stability and Proliferation in SCLC via c-MYC/PARP1 Suppression Driving Vulnerability to BET Inhibitors
Source: Research (Wash D C). 2025 Oct 2;8:0908. doi: 10.34133/research.0908 (PMC12489181; doi:10.34133/research.0908)
Supplement: Supplementary 1 — Figs. S1 to S8 Tables S1 to S4 [file research.0908.f1.zip › Supplementary tables 1-4 (revision).docx]

**Supplementary Table 1. siRNA sequences**

| siRNA name | Target sequence |
| --- | --- |
| c-MYC_1# | 5'-GCTTGTACCTGCAGGATCT-3' |
| c-MYC_2# | 5'-GAGGATATCTGGAAGAAAT-3' |
| c-MYC_3# | 5'-GGAAGAAATCGATGTTGTT-3' |
| siARID1A_1# | 5'-GCCCUGAACAAUAACCUCATT-3' |
| siARID1A_2# | 5'-CAGCUUGCCUGAUCUAUCUTT-3' |
| siPARP1_1# | 5'-CCGAGAAAUCUCUUACCUCAATT-3' |
| siPARP1_2# | 5'-ACGGUGAUCGGUAGCAACAAATT-3' |
| siNC | 5'-UUCUCCGAACGUGUCACGUTT-3' |

**Supplementary Table 2. DNA oligonucleotides used for shRNA studies**

| Gene name | shRNA targeting sequences |
| --- | --- |
| ARID1A 1# | 5'-CGTAATGACATGACCTATAAT-3' |
| ARID1A-2# | 5'-ACTGACTGTTGCCCTTTATTT-3' |
| shNC | 5'-AGTCTTAATCGCGTATAAGGC-3' |

**Supplementary Table 3. Primer Sequences for qRT-PCR**

| Primer name | Primer sequences |
| --- | --- |
| ARID1A | Forward: 5'-CAGTTCTCCACCCAAGGCAC-3' |
|  | Reverse: 5'-GCCGCTTGTAATTCTGCTGT-3' |
| c-MYC | Forward: 5'-AGGGAGATCCGGAGCGAATA-3' |
|  | Reverse: 5'-GTCCTTGCTCGGGTGTTGTA-3' |
| PARP1 | Forward: 5'-GAAATGCAGCGAGAGCATCC-3' |
|  | Reverse: 5'-CATCAAACATGGGCGACTGC-3' |
| RAD51 | Forward: 5’-CAACCCATTTCACGGTTAGAGC-3’ |
|  | Reverse: 5’-TTCTTTGGCGCATAGGCAACA-3’ |
| Actin | Forward: 5’-TCCCTGGAGAAGAGCTACGA-3’ |
|  | Reverse: 5’-AGCACTGTGTTGGCGTACAG-3’ |

**Supplementary Table 4. Primer Sequences for ChIP-PCR**

| Primer name | Primer sequences |
| --- | --- |
| c-Myc-P1 | Forward: 5′-AGAATAACAAGGAGGTGGCTGGAAAC-3′ |
|  | Reverse: 5′-CTACTGGCAGCAGAGATCATCGC-3′ |
| c-Myc-P2 | Forward: 5′-GGAACAGGCAGACACATCTCAGG-3′ |
|  | Reverse: 5′-GCACAGCTATCTGGATTGGATACCTT-3′ |
| PARP1 BS1 | Forward: 5’-ACCACAGCCTCAACCTTCAG-3’ |
|  | Reverse: 5’-TGAGCCCAGGAGTTCAAGAC-3’ |
| PARP1 BS2 | Forward: 5’-AAGTCGAGGTGGGAGGATTG-3’ |
|  | Reverse: 5’-TCGAAATTGTGGTAATGACTGCA-3’ |
