# Supplementary figures and images for: ARID1A Governs Genomic Stability and Proliferation in SCLC via c-MYC/PARP1 Suppression Driving Vulnerability to BET Inhibitors
Source: Research (Wash D C). 2025 Oct 2;8:0908. doi: 10.34133/research.0908 (PMC12489181; doi:10.34133/research.0908)

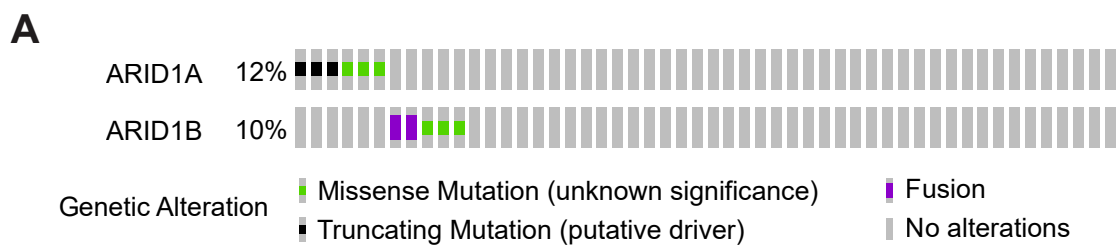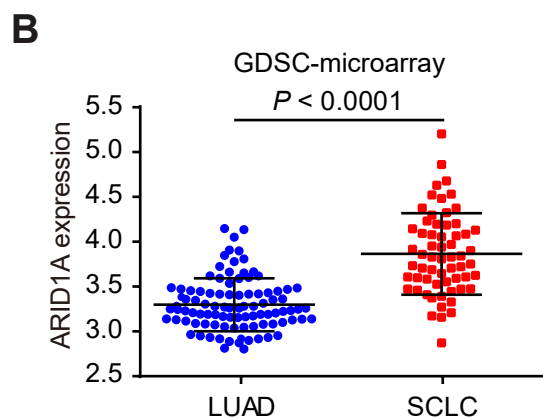

**Figure S1**

Supplement: Supplementary 1 — Figs. S1 to S8 Tables S1 to S4 [file research.0908.f1.zip › Supplementary Figure 1 (revision).pdf]

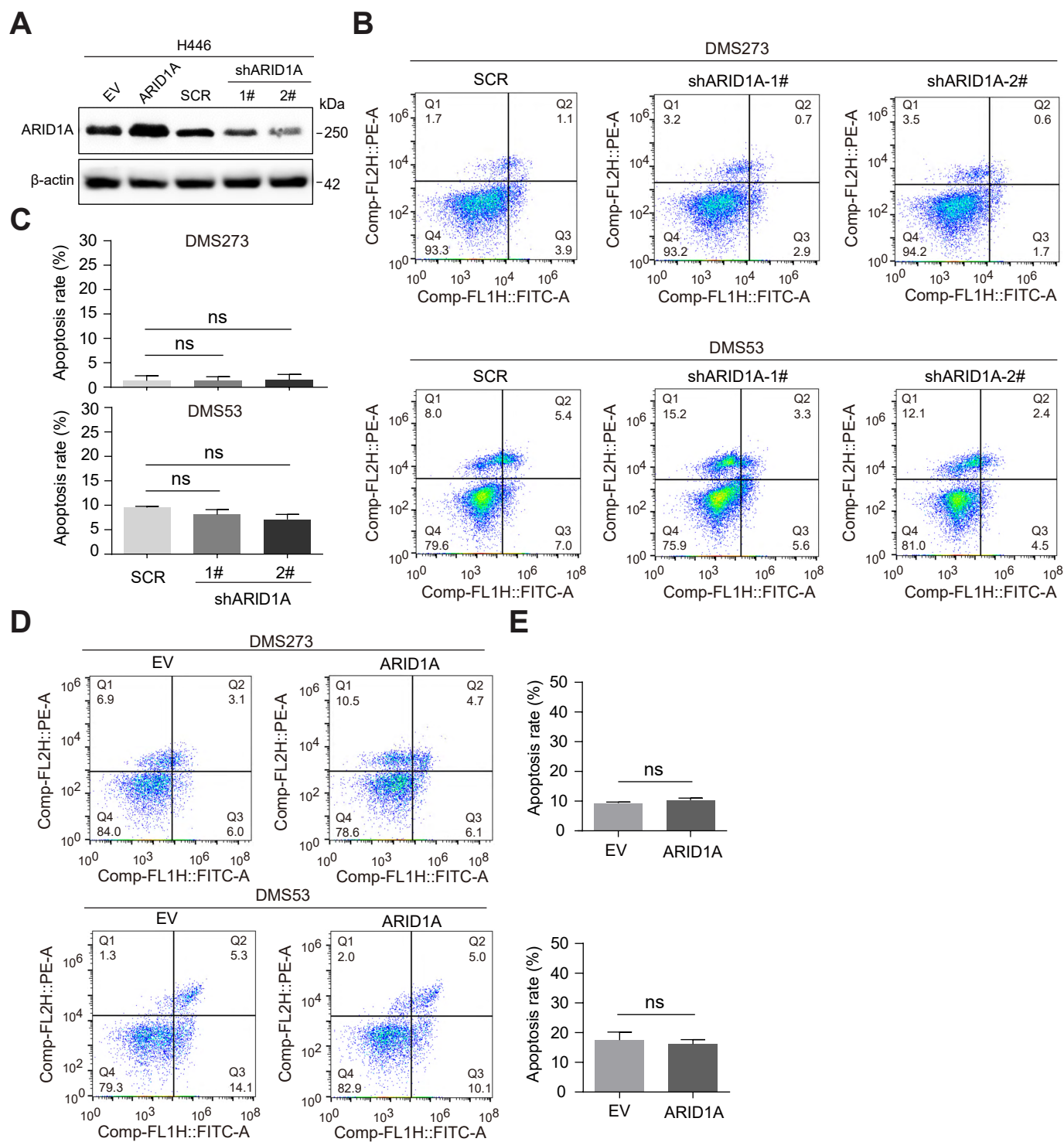

**Figure S2**

Supplement: Supplementary 1 — Figs. S1 to S8 Tables S1 to S4 [file research.0908.f1.zip › Supplementary Figure 2 (revision).pdf]

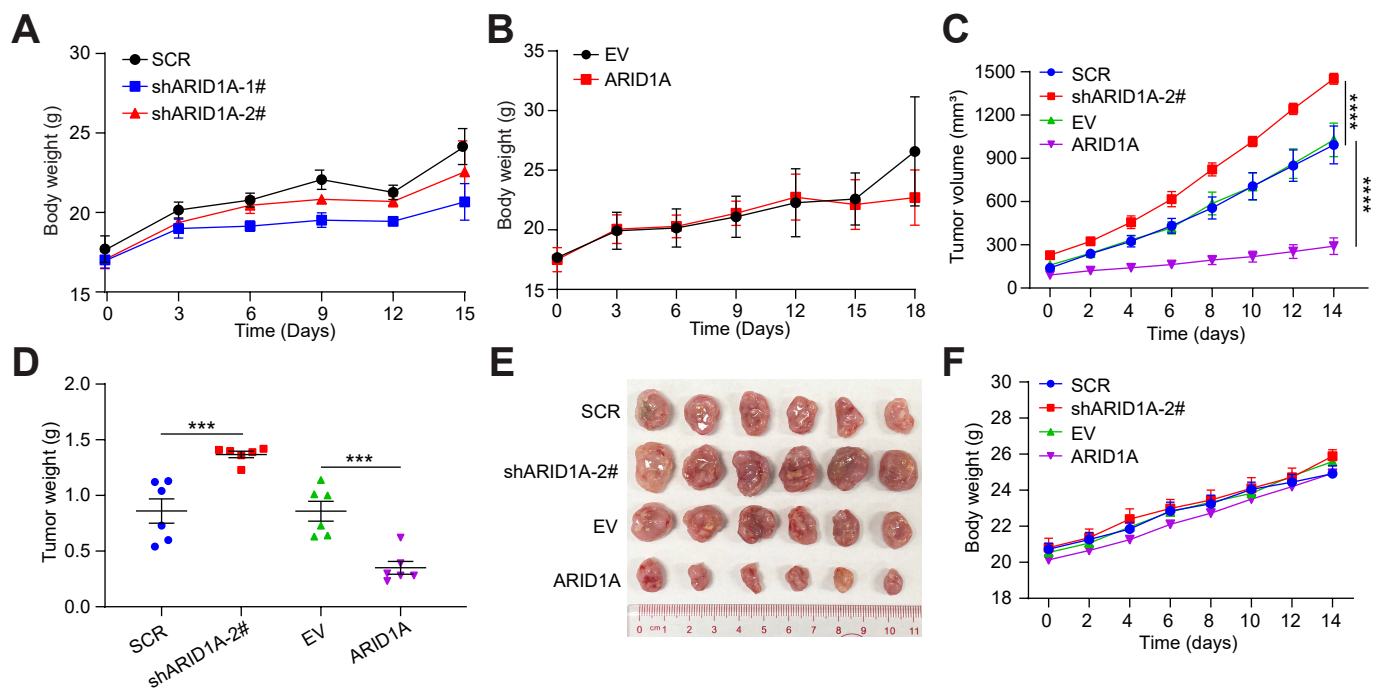

**Figure S3**

Supplement: Supplementary 1 — Figs. S1 to S8 Tables S1 to S4 [file research.0908.f1.zip › Supplementary Figure 3 (revision).pdf]

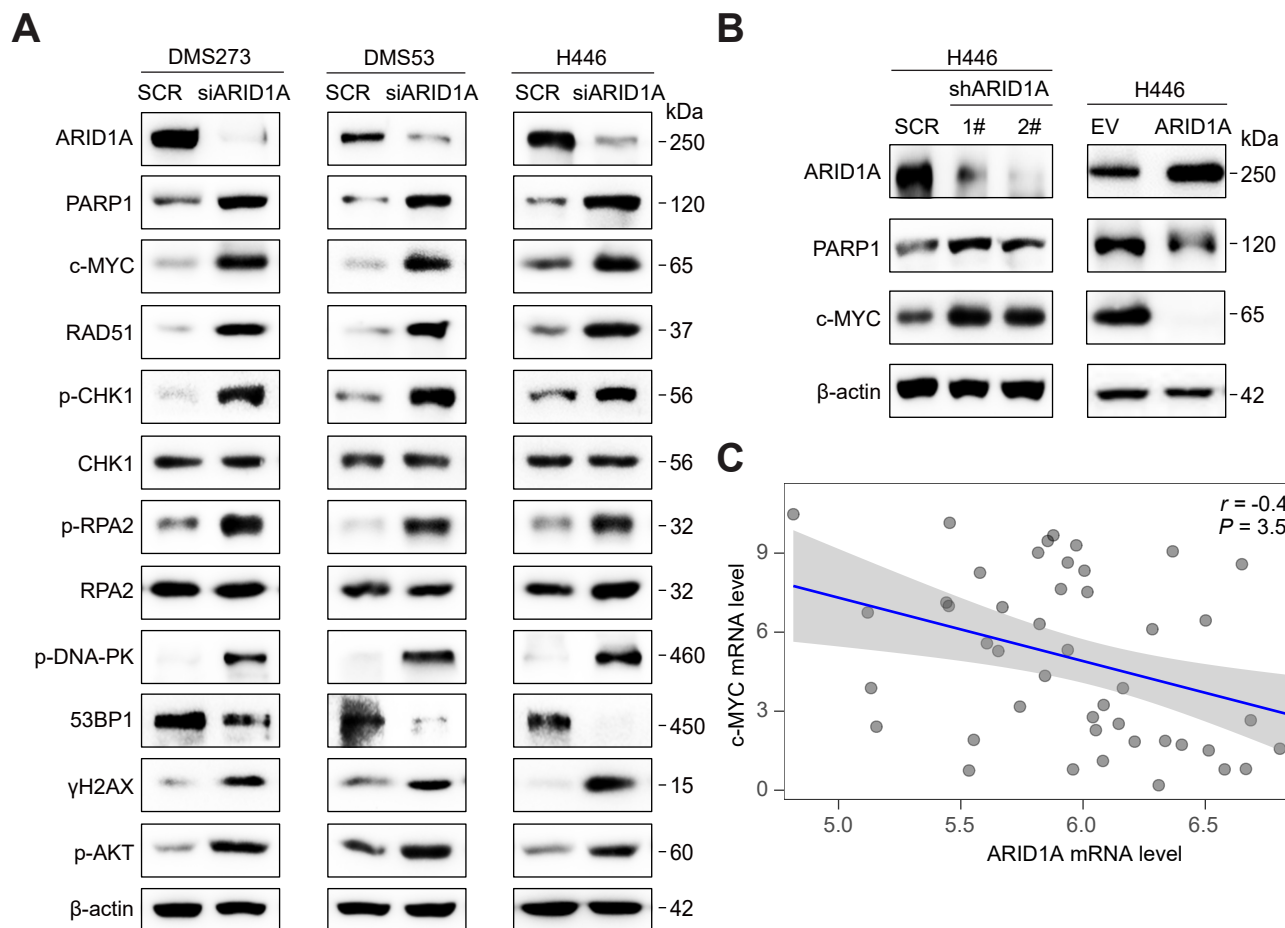

**Figure S4**

Supplement: Supplementary 1 — Figs. S1 to S8 Tables S1 to S4 [file research.0908.f1.zip › Supplementary Figure 4 (revision).pdf]

**A**

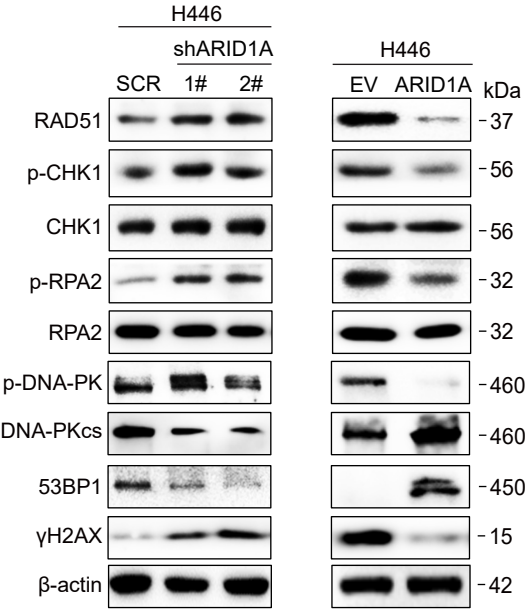

**Figure S5**

Supplement: Supplementary 1 — Figs. S1 to S8 Tables S1 to S4 [file research.0908.f1.zip › Supplementary Figure 5.pdf]

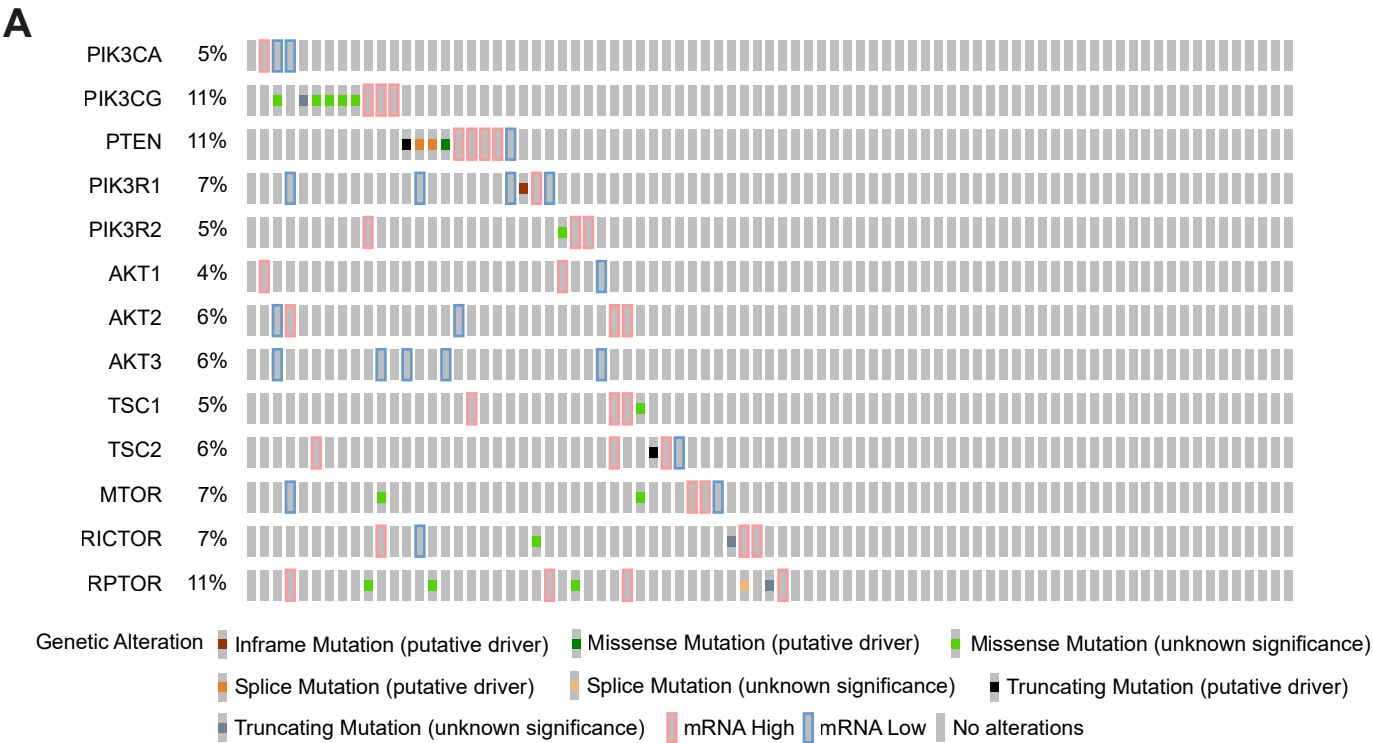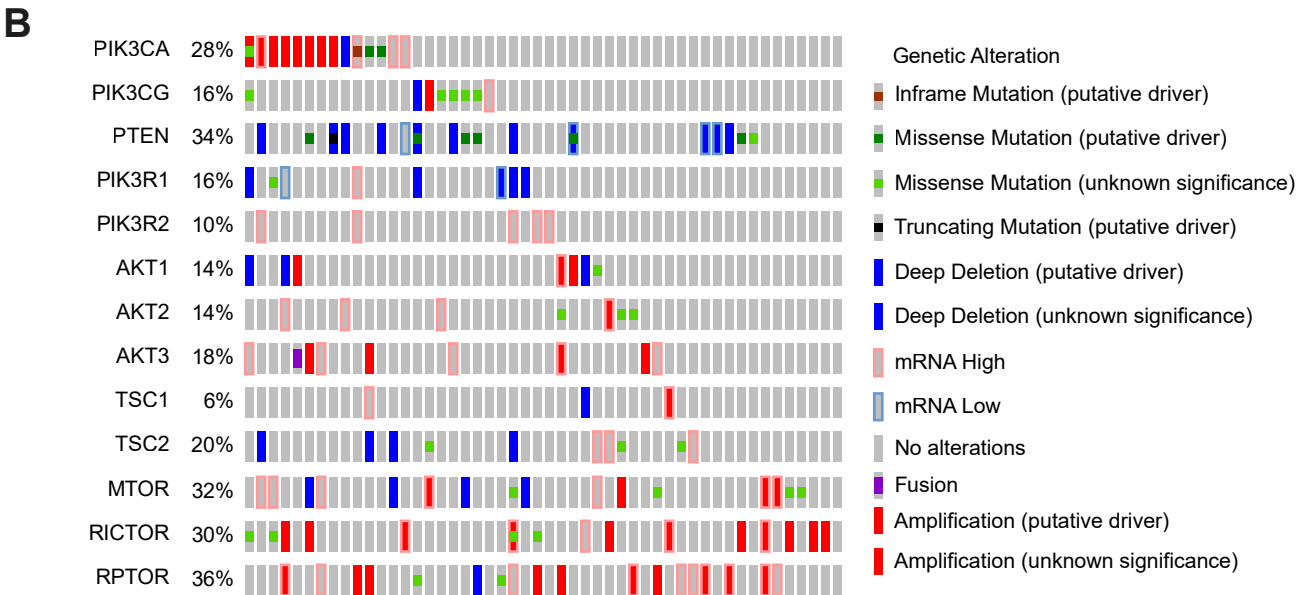

**Figure S6**

Supplement: Supplementary 1 — Figs. S1 to S8 Tables S1 to S4 [file research.0908.f1.zip › Supplementary Figure 6 (revision).pdf]

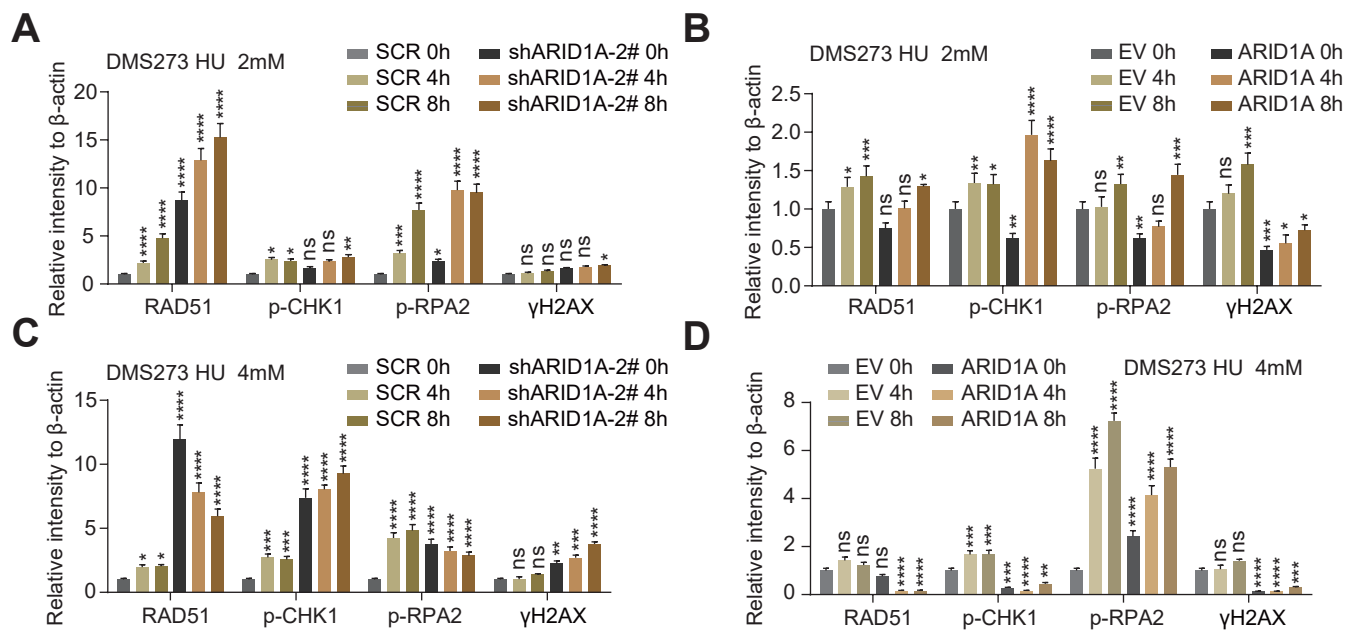

**Figure S7**

Supplement: Supplementary 1 — Figs. S1 to S8 Tables S1 to S4 [file research.0908.f1.zip › Supplementary Figure 7 (revision).pdf]

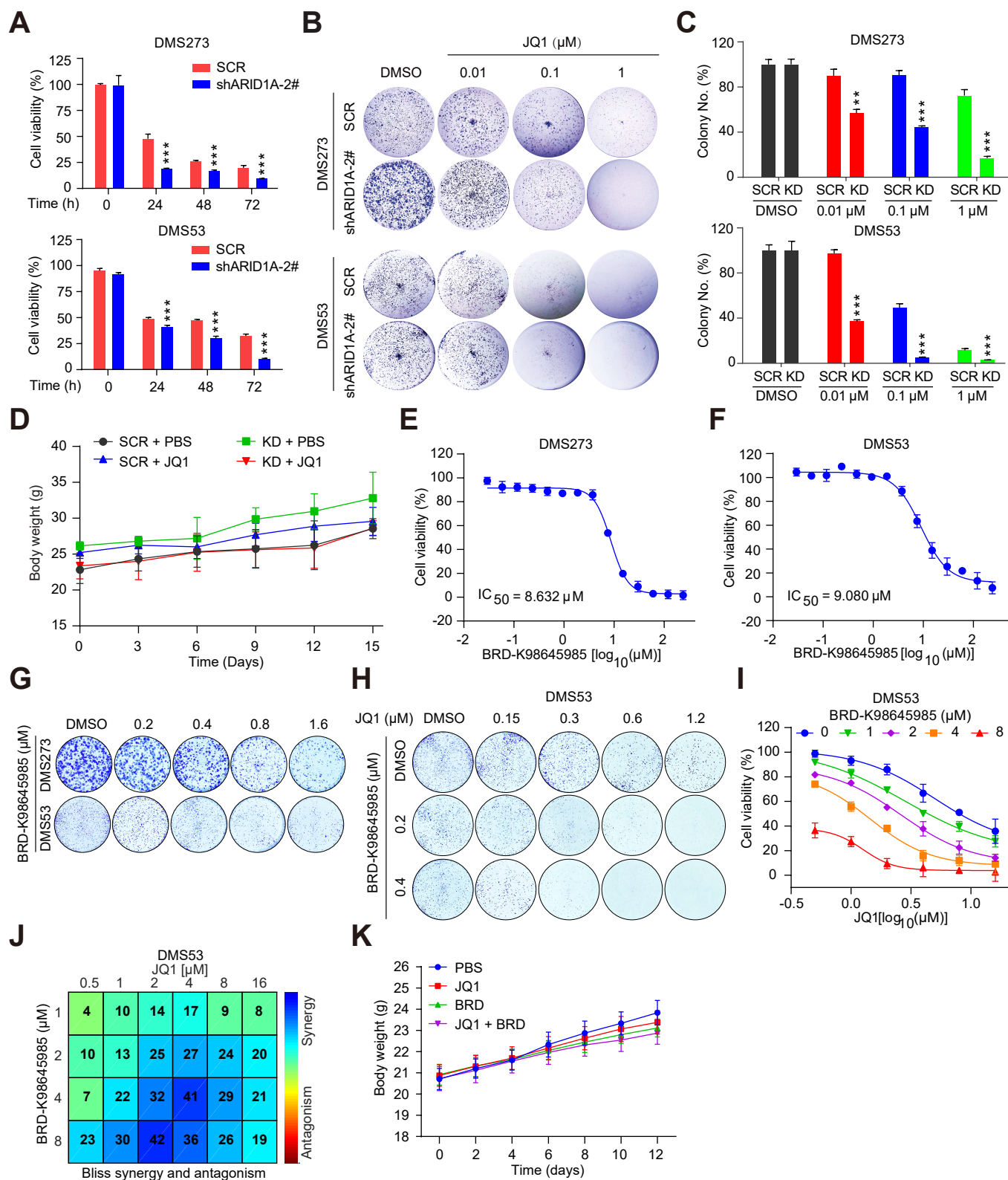

**Figure S8**

Supplement: Supplementary 1 — Figs. S1 to S8 Tables S1 to S4 [file research.0908.f1.zip › Supplementary Figure 8 (revision).pdf]
